# Production and immunogenicity of a deoxyribonucleic acid *Alphavirus* vaccine expressing classical swine fever virus *E2-Erns* protein and porcine *Circovirus Cap-Rep* protein

**DOI:** 10.3389/fmicb.2022.1065532

**Published:** 2022-12-06

**Authors:** Fuyu Du, Zhi Cao, Zixuan Ye, Jun He, Weijie Zhang, Ke Zhang, Pengbo Ning

**Affiliations:** ^1^School of Life Science and Technology, Xidian University, Xi’an, China; ^2^Shaoxing Academy of Biomedicine of Zhejiang Sci-Tech University, Shaoxing, China; ^3^College of Veterinary Medicine, Qingdao Agricultural University, Qingdao, China; ^4^Engineering Research Center of Molecular and Neuro Imaging Ministry of Education, School of Life Science and Technology, Xidian University, Xi’an, China

**Keywords:** DNA bivalent vaccine, CSFV, PCV2, coinfection, alphaviral plasmid

## Abstract

Classical swine fever virus (CSFV) and porcine *Circovirus* type 2 (PCV2) are economically pivotal infectious disease viruses of swine. Alphaviral RNA replicon plasmids have been used as an important vector for constructing nucleic acid vaccines. Here, we aimed to construct a recombinant alphaviral plasmid vaccine pSCA1-*E2-Erns-Cap-Rep* for the prevention and control of CSFV and PCV2. Our results showed that the recombinant alphaviral plasmid vaccine pSCA1-*E2-Erns-Cap-Rep* was successfully constructed. The vaccine encoding *E2* and *Erns* of CSFV, *Cap*, and *Rep* of PCV2 can induce E2, Erns, Cap, and Rep protein expression. ELISA analysis showed that mice-immunized pSCA1-*E2-Erns-Cap-Rep* plasmid vaccine produced higher anti–CSFV- and anti–PCV2-specific antibodies with dose- and time-dependent manners. Furthermore, neutralizing assays were measured using IF and ELISA methods. The results showed the production of neutralizing antibodies could neutralize CSFV (up to 2^10^.^13^) and PCV2 (2^8^.^6^) effectively, which exhibited the immune efficacy of the pSCA1-*E2-Erns-Cap-Rep* plasmid vaccine. Taken together, this pSCA1-*E2-Erns-Cp-Rep* plasmid vaccine could be considered a novel candidate vaccine against CSFV and PCV2.

## Introduction

Classical swine fever virus (CSFV) is a single-stranded positive-stranded enveloped RNA virus, belonging to the Flaviviridae genus *Pestivirus* (Tong et al., 2020). Classical swine fever (CSF) caused by CSFV is one highly contagious disease among swine, resulting in systemic hemorrhage in domestic pigs (Fan et al., 2020). At present, it is widespread in the world, though there are many regions that are free of CSF (Huang et al., 2009). Porcine *Circovirus* type 2 (PCV2) is one smallest non-enveloped single-stranded animal circular DNA viruses, which is distributed worldwide and has been illustrated as one of the most important agents of the postweaning multisystemic wasting syndrome among pigs (Allan and Ellis, 2000; Darwich et al., 2004).

Currently, coinfection and superinfection with CSFV and PCV2 are frequently found in domestic pigs, causing further challenges to the pig industry (Zhou et al., 2015). The disease caused by collaborative pathogenesis with CSFV and PCV2 is more severe and has higher mortality than infection by either virus alone posing a serious threat to pig health. During co-infection, the PCV2 infection plays a critical role related to the increase of PCV2-CSFV dual-positive cells, and CSFV infection usually is secondary to it (Zhou et al., 2015). When infected with CSFV, the number of IgM^+^, CD4^+^CD8^–^CD25^+^, CD4^+^CD8^+^CD25^+^, and CD4^–^CD8^+^CD25^+^ lymphocytes, as well as the neutralizing antibodies level of CSFV, was remarkably lower than that in the pigs co-infected with PCV2 and CSFV (Ouyang et al., 2019). Besides, PCV2 inhibits the proliferation of peripheral blood mononuclear cells (PBMCs) specifically induced by CSFV (Huang et al., 2011). The infection of an attenuated CSFV in macrophages was decreased in the PCV2 dose-dependent manner (Huang et al., 2014), which illustrated that PCV2 exerts an important role in the co-infection of CSFV and PCV2. These studies showed that PCV2 could improve the infection of CSFV by reducing the host immune response and inhibiting the efficacy of the CSFV vaccine. Therefore, co-infections of CSFV and PCV will be a public concern and attract more researchers’ attention.

Although the development of vaccines has played an important role in preventing and controlling CSF or PCVD, the co-infection or superinfection of CSFV and PCV has evidently increased difficulties in the prevention and control of diseases in the pig breeding industry in recent years (Ouyang et al., 2019). Some researchers have found that PCV2 could disturb the immune effect of the CSFV MLV vaccine (Huang et al., 2014; Chen et al., 2019). Besides, the protection efficacy of the CSFV LOM vaccine could also be affected by the infection of PCV2 (Lim et al., 2016). Confidently, PCV2-derived interference improved the invasion of CSFV in pigs and enhanced the difficulty of CSFV prevention (Huang et al., 2011). There are effective live-attenuated CSF vaccines available and inactivated and subunit vaccines available for PCV2.

Presently, DNA-based alphaviral RNA replicon plasmids have been employed as one key strategy for developing nucleic acid vaccines to eliminate the concerns for potential chromosomal integration and cell transformation generated by the use of naked DNA vaccines (Berglund et al., 1998; Leitner et al., 2000). Since the structural protein genes (nsP1–4) of the alphaviral RNA replicon plasmids did not express and produce progeny virus particles, only a short-term expression can be obtained, which eventually causes apoptotic cell death of transfected cells (Rayner et al., 2002). There has been reported that the Semliki Forest virus-derived suicidal DNA plasmid, pSCA1, was used for the HPV DNA vaccine development (Hsu et al., 2001). Therefore, the alphaviral RNA replicon plasmid vaccine was a better strategy for ensuring the safety of the host after immunization.

Classical swine fever virus glycoprotein E2 is one of the most important immunogenic proteins, (Zhang et al., 2018; Abid et al., 2019) and glycoprotein Erns (E0) of CSFV is an additional antigen that can be involved in protection against CSFV infection (Lin et al., 2004; Cheng et al., 2019). Both E2 and Erns are the targets for neutralizing antibodies and are involved in immune protection against CSFV (Li et al., 2016). The PCV2 genome encodes two important proteins, namely, Rep and Cap proteins. The Rep protein is related to the rolling circle replication of the viral genome. The Cap protein is the main antigenicity in which neutralizing epitope exists and is considered an important target for vaccine development (Wu et al., 2012). In this study, we aimed to develop a recombinant DNA vaccine pSCA1-*E2-Erns-Cap-Rep* for the prevention and control of the co-infection of CSFV and PCV2, to protect pigs from lethal infection. The *Alphavirus* plasmid pSCA1 was selected as the vector of *E2*, *Erns*, *Cap*, and *Rep*. Our data showed that the recombinant plasmid vaccine was successfully constructed, and it could express *E2, Erns, Cap*, and *Rep* protein *in vitro* and triggered the production CSFV and PCV2 antibody *in vivo*. These results could be considered as a novel strategy for vaccine development against CSFV and PCV2 co-infections in the future, providing a better understanding of the development of DNA bivalent vaccine.

## Materials and methods

### Preparation of recombinant plasmid pSCA1-*E2-Erns-Cap-Rep*

The nucleotide sequences of *E2*, *Erns*, *Cap*, and *Rep* were obtained from GenBank (National Center for Biotechnology Information, NCBI), which were synthesized on the pSCA1 plasmid in the Gene ray Biotechnology company. The length of *E2*, *Erns*, *Cap*, and *Rep* was 1,122, 681, 696, and 936 bp, respectively. Then, 100 μl of *Escherichia coli* strain DH5α was pre-cooled at −20°C for 30 min. Subsequently, the pSCA1 plasmid and *E. coli* strain DH5α were mixed for 2 min at 42°C and incubated at 37°C for 1.5 h, which would be used for the plasmid amplification. Sequencing was carried out by Gene Ray Biotechnology Company to support proper construction.

### Detection of *E2, Erns, Cap*, and *Rep* mRNA transcription level *in vitro*

PK15 cells and 3D4/21 macrophage cells were obtained from the American Type Culture Collection (Manassas, VA, USA). PK15 cells or 3D4/21 macrophage cells were, respectively seeded into a six-well plate and were cultured using Dulbecco’s modified Eagle medium (DMEM)/high-glucose medium (HyClone, USA) containing 10% fetal bovine serum (FBS) in an incubator at 37°C and 5% CO_2_. The number of seeding per well was 1 × 10^6^ cells. When the PK15 cell or 3D4/21 macrophage cell density reached 80%, the pSCA1-*E2-Erns-Cap-Rep* plasmid was transfected into cells, respectively. pSCA1-*E2-Erns-Cap-Rep* plasmid and Lipo2000 were added to 250 μL of serum-free OPTI-MEM medium, which was mixed and transferred to the six-well plate. Subsequently, 0, 12, and 24 h after transfection, PK15 cells or 3D4/21 macrophage cells were, respectively tested for the level *E2*, *Erns*, *Cap*, and *Rep* mRNA expression.

Subsequently, for RT-qPCR analysis, PK15 cells RNA and 3D4/21 macrophage cells RNA were, respectively extracted using the TRIzol method as previously described (Ning et al., 2016). The primers were designed using primer 5.0 software and are shown in Table 1. RNA was reverse-transcribed using PrimeScript™ RT reagent Kit (TAKARA) and measured the transcription level of *E2*, *Erns*, *Cap*, and *Rep* in PK15 cells or 3D4/21 macrophage cells through RT-PCR method using SuperReal PreMix Plus (SYBR Green) kit.

**TABLE 1:** Primer for RT-qPCR.

Primer	Sequence (5′→3′)
E2-F	TCCCCCGGGCGGCTAGCCTGCAAGGAAG
E2-R	CGCGGATCCACCAGCGGCGAGTTGTTCTG
Erns-F	TCCCCCGGGGAAAATATAACTCAATGGAACCTG
Erns-R	CGCGGATCCGGCATAGGCACCAAACCAG
Cap-F	GCATCTTCAACACCCGCCTA
Cap-R	ATCTCATCATGTCCACCGCC
Rep-F	TAGCCGAGCAGTTCCCTGTA
Rep-R TNF-α-F TNF-α-R IFN-γ-F IFN-γ-R	AGCTGTCTTCCAATCACGCT ATGAGCACTGAGAGCATGATCCG CCTCGAAGTGCAGTAGGCAGA CCTCAGATGTACCTAATGGTGG GCTTGATCACATCCATGCTCC

### Animal experiments

Specific pathogen-free (SPF) female BALB/c mice (age, 4–6 weeks; body weight, 20 ± 2 g) were obtained from the Air Force Medical University Animal Experimental Laboratory Center (Xi’an, China). Mice were housed at the animal house at 21 ± 1°C and with humidity at 55 ± 5%, respectively, with a 12-h light/dark cycle. The mice were randomly divided into seven groups (*n* = 4 mice/group) (Supplementary Figure 1). The mice model of DNA vaccine was established to determine whether the pSCA1-*E2-Erns-Cap-Rep* plasmid could produce CSFV-PCV2 antibody *in vivo*. Briefly, the animals were placed in the prone position and anesthetized with an intraperitoneal injection of 50 mg/kg pentobarbital sodium. Then, the pSCA1-*E2-Erns-Cap-Rep* plasmid of 50, 100, and 200 μg was subcutaneously injected into the right back and neck of mice. PBS was injected as naive control. In addition, we set up 50 μg + adjuvant, 100 μg + adjuvant, and 200 μg + adjuvant groups, in which we added 0.15 μL of 2 mol/L CaCl_2_, 100 μL of ddH_2_O, and 100 μL of 2 × Hebs, incomplete Freund’s adjuvant to each tube and mixed them with the plasmid to form one kind water-in-oil emulsifier to obtain the pSCA1-*E2-Erns-Cap-Rep* plasmid vaccine containing adjuvant. Then, the above vaccine containing adjuvant was injected subcutaneously into the right back and neck of mice. During the 3 days of observation after immunization with the vaccine, all mice survived the immunization without apparent signs of the disease and did not show any signs of distress. Subsequently, mice were anesthetized and blood samples were also collected from orbital venous, and serum samples were immediately frozen after separation and stored at −80°C. Blood was collected from the mice, and the serum was separated at 0-day post-immunization (dpi). Second immunization, third immunization, and fourth immunization were performed at 7, 14, and 21 dpi, respectively (Supplementary Figure 1). After blood samples were collected, mice were euthanized by CO_2_ asphyxiation at the termination of the experiments. All experimental procedures were approved by the Tab of Animal Experimental Welfare and Ethical Inspection.

### Determination of classical swine fever virus antibody and *Circovirus* type 2 antibody

The serum was obtained from the saline, 50 μg, 100 μg, 200 μg, 50 μg + adjuvant, 100 μg + adjuvant, and 200 μg + adjuvant groups at 0, 7, 14, 21, and 28 dpi, respectively. The levels of CSFV antibody and PCV2 antibody were measured using ELISA kits according to the manufacturer’s instructions, which were analyzed through the GraphPad prism 5.0 software (MSK Bio, Wuhan, China) (Karki et al., 2021).

### Determination of classical swine fever virus and *Circovirus* type 2 TCID_50_

To determine the TCID_50_ of CSFV and PCV2, 1 × 10^4^ PK15 cells were seeded into per well of 96-well plates and cultured in 5% CO_2_ and 37°C. Subsequently, 10-fold-diluted CSFV or PCV2 was added to PK15 cells for eight replicates. At the same time, the cell-negative group and virus-positive group were designed to calculate TCID_50_. The 96-well plates were incubated at 37°C for 2 h and washed five times with PBS. Then, DMEM medium supplemented with 2% FBS was added into each well with incubation at 37°C for approximately 5 days. The TCID_50_ of CSFV and PCV2 was measured using indirect immunofluorescence assay (IF) as previously described (Chen et al., 2015; Tables 2, 3).

**TABLE 2:** Fluorescence effects of Classical swine fever virus (CSFV)-infected PK15 cells during the 5-day observation period.

Dilution	Infections	No infections	Total		Percentage of infections (%)
			
			Infections	No infections	
10^–1^	8	0	33	0	100
10^–2^	8	0	25	0	100
10^–3^	8	0	17	0	100
10^–4^	5	3	9	3	75
10^–5^	3	5	4	8	33.3
10^–6^	1	7	1	15	6.25

**TABLE 3:** Fluorescence effects of *Circovirus* type 2 (PCV2)-infected PK15 cells during the 5-day observation period.

Dilution	Infections	No infections	Total		Percentage of infections (%)
			
			Infections	No infections	
10^–1^	8	0	39	0	100
10^–2^	8	0	31	0	100
10^–3^	8	0	23	0	100
10^–4^	7	1	15	1	93.75
10^–5^	5	3	8	4	66.67
10^–6^	2	6	3	10	23.08
10^–7^	1	7	1	17	5.56

### Determination of classical swine fever virus-neutralizing antibody

Serum samples obtained above (saline, 100 μg, 200 μg, 100 μg + adjuvant group, and 200 μg + adjuvant group) were heat inactivated for 30 min at 56°C and serially diluted two-fold (initial dilution concentration was 1:8) with three replicates for each concentration hole. Each diluted serum (50 μL) was mixed with 50 μL of 100 TCID_50_ CSFV, and the mixture was incubated at 37°C for 1 h. Subsequently, the single-layer PK15 cells (5 × 10^3^ cells per well) were infected using 100 μL of serum/serum mixtures, and the 96-well plate was incubated at 37°C and 5% CO_2_ atmosphere for about 5 days. Cells were fixed in 4% paraformaldehyde for 20 min after 5-day incubation and were then permeabilized with 0.3% Triton X-100 for 20 min at room temperature and blocked with 5% bovine serum albumin (BSA) for 1 h at 37°C. Subsequently, cells were incubated with CSFV anti-E2 protein antibody (diluted 1:500 in BSA) for 12 h at 4°C. The fixed cells were incubated with fluorescein isothiocyanate (FITC)-conjugated goat anti-rabbit (1:400 dilution, BioLegend) for 1 h at 37°C after washing three times using PBST. Cells were visualized using a fluorescence microscope (Leica) after nuclei staining with 4′,6-diamidino-2-phenylindole (DAPI) for 15 min at room temperature. Neutralizing antibody titers were determined as serum dilutions that fully protect 50% of cells from infection.

### Determination of *Circovirus* type 2 neutralizing antibody

Consistent with the treatment of serum samples described above, neutralizing antibody was tested using the ELISA method. Each diluted serum (5 μL) was mixed with 100 TCID_50_ PCV2 (5 μL), and mixtures were incubated at 37°C for 1 h. The OD_450 *nm*_ was measured to judge the neutralization efficacy against PCV2 using a commercial ELISA kit (MSKBIO) according to the manufacturer’s instructions.

### Detection of IFN-γ and TNF-α mRNA transcription level *in vivo*

After the immunization of the pSCA1-*E2-Erns-Cap-Rep* plasmid vaccine or PBS for 28 days, spleens were obtained from the PBS, 50 μg, 100 μg, 200 μg, 50 μg + adjuvant, 100 μg + adjuvant, and 200 μg + adjuvant groups, respectively. Subsequently, spleens were tested for the level of *IFN*-γ *and TNF*-α mRNA expression. The primers are shown in Table 1. RNA was reverse-transcribed using PrimeScript™ RT reagent Kit (TAKARA). The transcription level of *IFN*-γ *and TNF*-α in spleens of mice after immunization was measured through the RT-PCR method using SuperReal PreMix Plus (SYBR Green) kit according to the manufacturer’s instructions, which were analyzed through 2^–ΔΔ*CT*^ method.

### Statistical analysis

Data were presented as the mean ± standard deviation (SD) of three independent experiments. Statistical comparisons were analyzed by the one-way analysis of variance (ANOVA) using SPSS 23.0 software (SPSS Inc., Chicago, IL, USA). *P < 0.05 was considered statistically significant.

## Results

### Construction of recombinant plasmid pSCA1-*E2-Erns-Cap-Rep*

**FIGURE 1:**
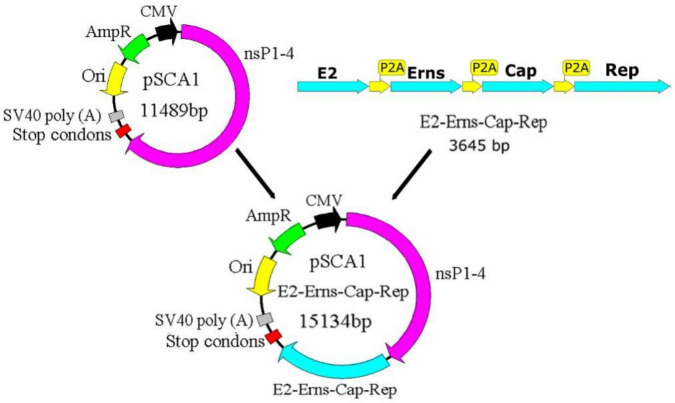
Diagram representation of the recombinant plasmid pSCA1-*E2-Erns-Cap-Rep*. CMV is the eukaryotic promoter. Stop codons are the termination signal. SV40 poly (A) is the strong transcription termination signal, which could normally terminate transcription and further enhance its stability. The ampicillin resistance is for selection and maintenance in *Escherichia coli*. NsP1–4 are nsP1, nsP2, nsP3, and nsP4, which were four non-structural proteins and play important role in RNA synthesis. CMV, cytomegalovirus; Amp, ampicillin; Ori, origin site; nsP1–4, non-structural proteins 1–4; SV40 poly (A), Simian virus 40 polyadenylic acid A.

The constructed recombinant plasmid pSCA1-*E2-Erns-Cap-Rep* is illustrated schematically in Figure 1. *E2, Erns, Cap, and Rep* could be expressed using the pSCA1-*E2-Erns-Cap-Rep* plasmid. Cleavage follows the disulfide cyclic peptide sequence and is performed by the P2A sequence with a size of 66 bp in the *E2-Erns-Cap-Rep* fragment whose production contains cleavable protein. The recombinant plasmid pSCA1-*E2-Erns-Cap-Rep* was verified as being correct *via* sequencing. The sequencing results showed that there was no mutation in the constructed *E2-Erns-Cap-Rep* fragment sequence during the construction process, illustrating that the *E2-Erns-Cap-Rep* segment was inserted into the plasmid pSCA1 and the pSCA1-*E2-Erns-Cap-Rep* plasmid was successfully constructed (refer to Supplementary File 1).

### pSCA1-*E2-Erns-Cap-Rep* increases *E2, Erns, Cap*, and *Rep* mRNA levels *in vitro*

**FIGURE 2:**
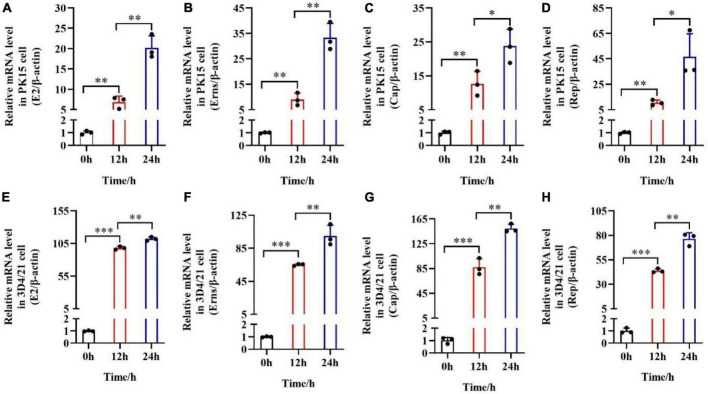
mRNA expression levels of *E2, Erns, Cap*, and *Rep in vitro* after the transfection of pSCA1-*E2-Erns-Cap-Rep*. **(A–D)** The mRNA level of *E2, Erns, Cap*, and *Rep* was normalized based on β*-actin* expression in PK15 cells, respectively. **(E–H)** The mRNA level of *E2, Erns, Cap*, and *Rep* was normalized based on β*-actin* expression in 3D4/21 macrophage cells, respectively. Three independent qPCR experiments always obtained consistent conclusion, and one of the results was shown. * denotes the significant difference between different groups (*P < 0.05, **P < 0.01, and ***P < 0.001).

After the pSCA1-*E2-Erns-Cap-Rep* plasmid was transfected into PK15 cells or 3D4/21 macrophage cells, *E2*, *Erns*, *Cap*, and *Rep* primers were used to detect the mRNA expression of these genes in PK15 cells and 3D4/21 macrophage cells, respectively. Successful expression of *E2, Erns, Cap*, and *Rep* was confirmed in transfected PK15 cells or 3D4/21 macrophage cells by RT-qPCR analysis (Figure 2). The upregulation of *E2*, *Erns*, *Cap*, and *Rep* was significantly improved in the PK15 cells transfected with the pSCA1-*E2-Erns-Cap-Rep* plasmid in 12 h compared with those of the PK15 cells transfected with the plasmid in 0 h (**P < 0.01). Besides, the expression level of *E2*, *Erns*, *Cap*, and *Rep* mRNA was remarkably increased in PK15 cells transfected with the pSCA1-*E2-Erns-Cap-Rep* plasmid in 24 h compared with those of PK15 cells transfected with the plasmid in 12 h (**P < 0.01). Meaningfully, a consistent finding was found when it was carried out with 3D4/21 macrophage cells (Figure 2). Their results indicated that the pSCA1-*E2-Erns-Cap-Rep* plasmid could enhance the upregulation of *E2*, *Erns*, *Cap*, and *Rep* mRNA in host cells.

### pSCA1-*E2-Erns-Cap-Rep* plasmid vaccine elicited classical swine fever virus and *Circovirus* type 2-specific antibodies

**FIGURE 3:**
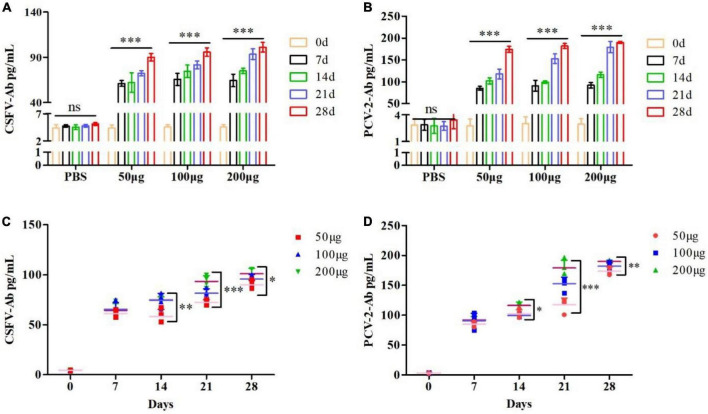
Evaluation of pSCA1-*E2-Erns-Cap-Rep* plasmid vaccine immunogenicity. **(A,B)** CSFV and PCV2 antibody assay of pSCA1-*E2-Erns-Cap-Rep* plasmid in serum of mice among 50, 100, and 200 μg groups, respectively, were determined by ELISA assay after immunization at different times. **(C,D)** The production of CSFV and PCV2 antibodies by the pSCA1-*E2-Erns-Cap-Rep* plasmid vaccine after immunization was compared with different doses. * denotes the significant difference between the pSCA1-*E2-Erns-Cap-Rep* plasmid vaccine group (50, 100, and 200 μg groups) and PBS group (*P < 0.05, **P < 0.01, and ***P < 0.001). All data are represented as mean ± SD (*n* = 4).

To further investigate whether the pSCA1-*E2-Erns-Cap-Rep* plasmid vaccine could express the *E2, Erns, Cap*, and *Rep* protein and elicit specific antibody responses, we, respectively detected the CSFV antibody and PCV2 antibody level of immunized serum. The results showed that the CSFV and PCV2 antibody levels of pSCA1-*E2-Erns-Cap-Rep* plasmid vaccine groups (50, 100, and 200 μg groups) were significantly higher than those in the saline group at 7, 14, 21, and 28 days (*P* < 0.05), which illustrated that pSCA1-*E2-Erns-Cap-Rep* could stimulate mice to generate CSFV and PCV2 antibodies (Figures 3A,B). The highest CSFV and PCV2 antibody levels of vaccine groups were found at 28 dpi, and a time-dependent manner on antibody responding was addressed.

Subsequently, the dose-dependent pSCA1-*E2-Erns-Cap-Rep* plasmid vaccine was compared among different vaccine groups, respectively. Statistically significant differences in both the CSFV and PCV2 antibody levels were found among different doses of vaccine groups at 14, 21, and 28 dpi (*P* < 0.05), except that of 7 days was not found, indicating that the pSCA1-*E2-Erns-Cap-Rep* plasmid vaccine improved the levels of CSFV and PCV2 antibody in a dose-dependent manner, especially in the late timespoints (Figures 3C,D).

### Adjuvant enhanced the immunization effect of the pSCA1-*E2-Erns-Cap-Rep* plasmid vaccine

**FIGURE 4:**
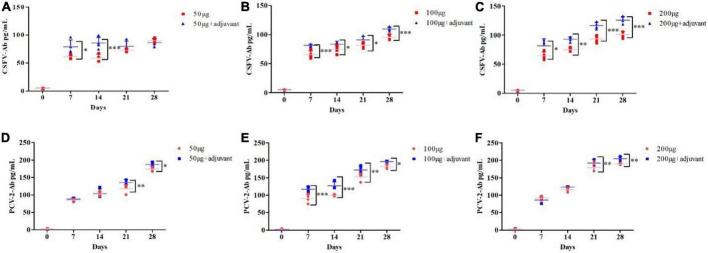
Adjuvants enhanced the CSFV and PCV2 antibody levels after pSCA1-*E2-Erns-Cap-Rep* plasmid vaccine immunization *in vivo*. **(A–C)** Compared with the 50, 100, and 200 μg pSCA1-*E2-Erns-Cap-Rep* plasmid vaccine group, the 50, 100, and 200 μg + adjuvant groups, respectively, stimulated the increase of CSFV antibody levels in mice; **(D–F)** Compared with the 50, 100, and 200 μg pSCA1-*E2-Erns-Cap-Rep* plasmid vaccine group, the 50, 100, and 200 μg + adjuvant groups, respectively, stimulated the increase of PCV2 antibody levels in mice. *, **, and *** denote the significant difference between different groups (*P < 0.05, **P < 0.01, and ***P < 0.001). All data are represented as mean ± SD (*n* = 4).

To further improve the immune efficacy of the pSCA1-*E2-Erns-Cap-Rep* plasmid vaccine, incomplete Freund’s adjuvant (IFA) was used to form water-in-oil vaccine and the 50 μg, 100 μg, and 200 μg groups were compared with 50 μg + adjuvant, 100 μg + adjuvant, and 200 μg + adjuvant groups, respectively. The results showed that the level of CSFV antibody was remarkably higher in 50 μg + adjuvant (7 and 14 dpi), 100 μg + adjuvant, and 200 μg + adjuvant groups than those in 50 μg, 100 μg, and 200 μg groups (*P* < 0.05). The level of PCV2 antibody was significantly lower in 50, 100, and 200 μg groups at 21 and 28 dpi than those in 50 μg + adjuvant, 100 μg + adjuvant, and 200 μg + adjuvant groups (*P* < 0.05), respectively. Those results indicated that higher CSFV antibody and PCV2 antibodies were induced and the adjuvant improved the immune efficacy of the pSCA1-*E2-Erns-Cap-Rep* plasmid vaccine (Figure 4).

### Neutralizing antibody responses following immunization with pSCA1-*E2-Erns-Cap-Rep*

**FIGURE 5:**
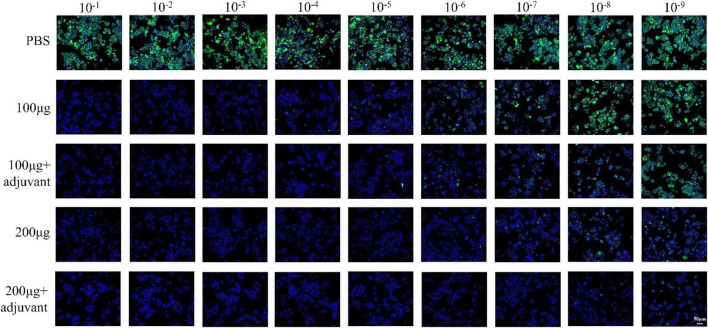
Determination of the neutralized CSFV effect of the pSCA1-*E2-Erns-Cap-Rep* plasmid vaccine. Representative immunofluorescence images of PK15 cells infected the remaining CSFV neutralized by neutralizing antibody stained with nucleus (DAPI) and CSFV (green).

**FIGURE 6:**
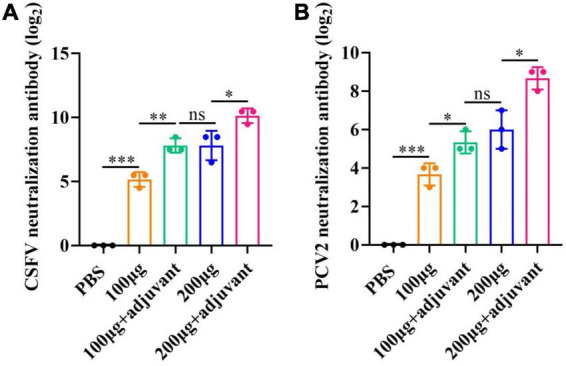
Viral neutralizing responses determination post-vaccination. **(A)** Neutralization assay using indirect immunofluorescence for CSFV. **(B)** Neutralization assay using ELISA for PCV2. Significance is presented as *p < 0.05, **p < 0.01, and ***p < 0.001. Error bars represent SD.

To further investigate the immune efficacy of the pSCA1-E2-Erns-Cap-Rep plasmid vaccine, the neutralizing antibodies against CSFV (10^3^.^4^TCID_50_/mL) (Supplementary Figure 2) and PCV2 (10^4^.^38^TCID_50_/mL) (Supplementary Figure 3) viruses were assessed in immunized serum (saline, 100 μg, 200 μg, 100 μg + adjuvant group, and 200 μg + adjuvant group) (Figure 5). The results showed that the virus-neutralizing antibodies were detected in serums of 100 μg, 200 μg, 100 μg + adjuvant, and 200 μg + adjuvant groups; however, it was not found in the saline group serum. It was noted that the serums of the 100 μg group, 200 μg group, 100 μg + adjuvant groups, and 200 μg + adjuvant groups could neutralize CSFV effectively, in which the range of neutralizing antibody titer was 2^5^.^16^–2^10^.^13^. Moreover, the neutralizing effect of the 100 μg + adjuvant group and 200 μg + adjuvant group was significantly better than that of the 100 μg group and 200 μg group. Interestingly, in the further detection of viral neutralization activity against PCV2, similar results were obtained for PCV2 neutralizing antibodies by ELISA. The neutralizing antibody was detected as negative in the saline group; however, the titers of neutralizing antibodies exhibited a remarkable increase in all vaccinated groups. The amount of unnaturalized virus gradually decreased with dose increasing among the 100 μg group (2^3^.^7^), 200 μg group (2^6^), 100 μg + adjuvant group (2^5^.^3^), and 200 μg + adjuvant group (2^8^.^6^), and the highest antibody titer could reach up to 2^8^.^6^. These results indicated that the increase in the pSCA1-*E2-ERNS-Cap-Rep* plasmid vaccine dose could stimulate the production of neutralizing antibodies with a high titer, and the adjuvant could further improve the immune efficacy of the vaccine and enhance the ability of virus neutralization (Figure 6).

### pSCA1-*E2-Erns-Cap-Rep* plasmid vaccine stimulates the production of IFN-γ and TNF-α *in vivo*

**FIGURE 7:**
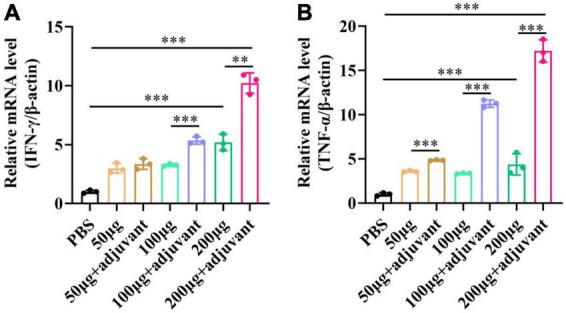
The expression level of *IFN*-γ and *TNF*-α after the pSCA1-*E2-Erns-Cap-Rep* plasmid vaccine immunization in spleens. **(A)** The *IFN*-γ was consistently increased with the increasing dose of plasmid vaccine with adjuvant and no adjuvant groups. **(B)** The *TNF*-α was consistently increased with the increasing dose of plasmid vaccine with adjuvant and no adjuvant groups. Three independent qPCR experiments always obtained consistent conclusions and one of the results was shown. * denotes the significant difference between different groups (**P < 0.01 and ***P < 0.001).

pSCA1-*E2-Erns-Cap-Rep* plasmid vaccine or PBS was injected through the subcutaneous (s.c.) manner. After 28 days, spleens were obtained from seven groups, which were used to determine the mRNA level of cytokines *IFN*-γ and *TNF*-α (Figure 7). The results showed that *IFN*-γ and *TNF*-α levels gradually increased in a dose-dependent manner among different vaccine groups without and with adjuvant, respectively. The level of *IFN*-γ and *TNF*-α was remarkably higher in 100 μg and 200 μg adjuvant + plasmid vaccine groups than those in vaccine groups (*P* < 0.05). These results illustrated that the Semliki Forest virus replicon-vectored DNA vaccine expressing the *E2, Erns, Cap*, and *Rep* protein of CSFV-PCV2 induced cell-mediated immune responses in mice, and the adjuvant enhanced these cell-mediated immune responses.

## Discussion

Although many measures are used for the prevention and control of CSFV and PCV2, these diseases are widespread around the world (Lei et al., 2016). It is known that co-infections derived from diverse viruses of livestock, such as CSFV and PCV2, can be found in the herds, leading to more severe concerns (Huang et al., 2011; Ouyang et al., 2019). At present, there were many commercial conventional live-attenuated vaccines for CSFV, including Pestiffa (French), VADIMON (USA), and Riems (Germany). However, these conventional monovalent vaccines could not prevent the co-infection of CSFV and PCV2 (Lim et al., 2016). Furthermore, despite the subunit vaccines of CSFV or PCV2 have been developed using recombinant CSFV E2 proteins or PCV2 Cap protein, their immunogenicity and protection effect were less than traditional live-attenuated vaccines (Wu et al., 2012; Zhang et al., 2018; Abid et al., 2019). In terms of the impact of co-infection on vaccine protection, there were different reports. Lim et al. (2016) examined the effect of PCV2 alone and PCV2-PRRSV co-infection on the potency of the LOM (CSF vaccines, LOM strain, widely used in Korea) vaccine in pigs and found that the efficacy of LOM CSF vaccines was not affected by the infection of PRRSV or/and PCV2 in Korea. However, Huang et al. (2011) reported that PCV2 infection in 2011 could decrease the efficacy of the LPC vaccine, which is an attenuated strain of CSFV in Taiwan. We considered that these phenomena are attributed to the complex pathological mechanism of co-infection and the different immunity mechanisms of different vaccines. Thus, it is important to research a kind of novel bivalent vaccine against CSFV and PCV2 co-infections.

Deoxyribonucleic acid vaccines have been shown to induce desirable immune responses and exhibit unbeatable advantages over traditional vaccines (Gerdts et al., 1997), but their immune efficacy is generally lower than that of conventional vaccines (Tacket et al., 1999). However, SFV replicon-based vector pSCA1 resulted in the lysis of transfected cells, which eliminated the concerns of genome integration of hosts and viruses (Cheng et al., 2001; Wang et al., 2015). To date, Semliki Forest virus replicon-vectored DNA vaccines have become one key strategy in vaccine development, successfully enhancing immunogenicity and improving the biosafety of conventional DNA vaccines (Berglund et al., 1998). We reasoned that the pSCA1 vector would provide an attractive platform for designing vaccines to tackle the co-infection or superinfection of CSFV and PCV.

*E2* and *Erns* proteins are the main structural proteins of CSFV, which could stimulate the body to produce neutralizing antibodies, thereby forming immune protection. *Cap* and *Rep* proteins are the main immune protein in PCV2, inducing the host to produce neutralizing antibodies. Similarly, Li et al. (2007) constructed a DNA alphaviral vector vaccine expressing CSFV-E2 by which neutralizing antibodies were induced successfully, while Park et al. (2017) constructed a DNA vaccine expressing PCV2 *Cap* by which PCV2 and diluted serums were mixed and incubated into PK15 cells to measure the neutralizing antibodies. The viral 2A peptide already, “self-cleaving” peptide, was used to mediate the expression of polycistronic in the aspect of gene therapy (Szymczak et al., 2004; Okita et al., 2008; Carey et al., 2009), which could be cleaved at 2A peptide C terminus during protein translation. Several studies have illustrated that the expression of multiple transgenes was more efficient than IRES (Szymczak et al., 2004; Chinnasamy et al., 2006; Okita et al., 2008; Carey et al., 2009). Therefore, in this study, we designed the recombinant plasmid pSCA1-*E2-Erns-Cap-Rep*, which added the 2A peptide among genes and used it as the DNA vaccine against the co-infection of CSFV and PCV2.

To evaluate whether *E2, Erns, Cap*, and *Rep* could be expressed by the plasmid pSCA1-*E2-Erns-Cap-Rep*, we found that the recombinant plasmid pSCA1-*E2-Erns-Cap-Rep* could express in the PK15 cells and 3D4/21 macrophage cells. It was consistent with the results of the previous report (Wang et al., 2015), indicating that the recombinant plasmid could work *in vitro*. Subsequently, to measure whether the pSCA1-*E2-Erns-Cap-Rep* plasmid vaccine has immunogenicity *in vivo*, we used the mice model and found that the pSCA1-*E2-Erns-Cap-Rep* plasmid vaccine could stimulate the production of CSFV and PCV2 antibody *in vivo*, as well as a similar increase, was observed in cell-mediated immune responses when compared to the PBS group. It is known that the antibody level is consistent with the level of IFN-γ and TNF-α cytokines in the host. IFN-γ has the function of cell-regulating cytokines, whereas TNF-α has a variety of biological functions, including the activation of innate immune memory (Reddy et al., 2001; Wang et al., 2015). The testing results showed that the present vaccine can induce cellular immune responses in mice. These results may be related to some studies which reported that the double-stranded RNA intermediate product produced by SFV replicon-based vector pSCA1-mediated expression can be used as an adjuvant for specific stimulation of antigen-encoding T cells (Wang et al., 2015). Therefore, the cellular immune response induced by the pSCA1-*E2-Erns-Cap-Rep* plasmid may play an important role in the immunization progress *in vivo*.

In this present study, different doses of pSCA1-*E2-Erns-Cap-Rep* plasmid vaccine groups induced higher CSFV and PCV2 antibodies than the negative control groups (*P* < 0.05). In addition, some techniques could be used to improve the immune effect of SFV replicon-based DNA vaccines, such as adding an adjuvant strategy (Hsu et al., 2001; Zhao et al., 2009). Incomplete Freund’s adjuvant (IFA), a water-in-oil emulsion adjuvant, has been widely used in veterinary vaccine products and tested in humans (Jensen et al., 1998; Billiau and Matthys, 2001) and enhances the immune efficacy significantly by prolonging the duration of antigen persistence (O’Hagan and De Gregorio, 2009). IFA contributes to the cell recruitment and induction of different cell immune types to uptake antigens and increases endogenous inflammatory cytokines, such as IFN-γ and TNF-α. Also, IFA can promote inflammation and initiate the innate and acquired immune response by establishing an immunocompetent environment to process and present antigens (Vitoriano-Souza et al., 2012). Besides, IFA induces a preferable Th2 type response mainly, and defective cellular response limits its adjuvant activity (Luo et al., 2012). It was previously reported that when IFA was used alone, IgG1 antibody and IL-4 production were higher relatively (Luo et al., 2012). However, mixing or using IFA in conjunction with other adjuvant-active compounds has been successful in animal testing and preclinical trials (Jensen et al., 1998). Hence, the plasmid vaccine was optimized by adding an adjuvant using IFA. After adding the IFA, we found that the IFA significantly improved the immune effect of the pSCA1-*E2-Erns-Cap-Rep* DNA vaccine, which is consistent with Pollack’s results that IFA enhancing systemic immune responses (Pollack et al., 2020).

To further evaluate the activity of neutralizing antibodies, we performed serum efficacy studies to demonstrate neutralization activity against PCV2 and CSFV. The method used in this study was to dilute neutralizing antibodies serum with the fixed virus. The serum was sequentially diluted two-fold and incubated with a certain amount of virus (100 TCID_50_). CSFV-neutralizing antibodies were measured by indirect immunofluorescence. Neutralizing antibody titers are the inverse of the serum dilution that protects 50% of cells from viral infection. The results showed that different dose groups could produce different titers of neutralizing antibodies to neutralize CSFV. Furthermore, the pSCA1-*E2-Erns-Cap-Rep* plasmid vaccine elicited high neutralizing antibody titers against CSFV in mice, and the highest dose group reached 2^10^.^13^ with better immune effects, which was consistent with the previous study (Lin et al., 2012; Ding et al., 2021). In addition, we used a commercial ELISA kit to detect the content of PCV2 Cap protein in the serum after virus neutralization. Subsequently, the antibody titer was calculated, and different doses of pSCA1-*E2-Erns-Cap-Rep* plasmid vaccine groups could stimulate high neutralizing antibody titers against PCV2, up to 2^8^.^6^ in the highest dose group, indicating that the vaccine can produce better neutralizing antibodies. In this research, we used IF and ELISA methods to determine the TCID_50_ of CSFV and PCV2 and neutralizing antibody titer, which provided a referable approach for those viruses without CPEs, and the desired results have been obtained. The evaluation and optimization in pigs for immunogenicity and protection against experimental infections of CSFV and PCV2 will be undertaken to develop a commercial vaccine in the future. The DNA *Alphavirus* vaccine will be used to immunize newborn piglets in the subsequent study, and it is expected that the DNA vaccine could provide dual protection for piglets against PCV2 and/or CSFV infection, achieving the dual prevention aim of a DNA *Alphavirus* vaccine.

In conclusion, an SFV replicon-based recombinant plasmid pSCA1-*E2-Erns-Cap-Rep* DNA vaccine encoding the *E2, Erns*, *Cap*, and *Rep* protein could successfully express proteins in PK15 cell and porcine alveolar macrophage *in vitro*, as well as immune responses in a mouse model. Therefore, the novel pSCA1-*E2-Erns-Cap-Rep* plasmid DNA vaccine is a strong candidate for an effective DNA vaccine against co-infection of CSFV and PCV2. Our study may help to provide a novel strategy for the development of candidate vaccines to tackle co-infection or superinfection of CSFV and PCV2.

## Data availability statement

The datasets presented in this study can be found in online repositories. The names of the repository/repositories and accession number(s) can be found in the article/Supplementary material.

## Ethics statement

This animal study was reviewed and approved by the Institutional Animal Care and Use Committee of the Fourth Military Medical University.

## Author contributions

FD, ZC, and ZY: data curation. JH and WZ: formal analysis. FD, ZC, ZY, and JH: methodology. PN: project administration and writing—review and editing. ZC and PN: supervision. FD, ZC, and KZ: writing—original draft. All authors contributed to the article and approved the submitted version.

## Supplementary material

Supplementary Figure 1 Experimental design to assess the CSFV-PCV2 antibody production of pSCA1-*E2-Erns-Cap-Rep* plasmid *in vivo*. BALB/c mice were subcutaneously injected with pSCA1-*E2-Erns-Cap-Rep* plasmid 50 μg group, 100 μg group, 200 μg group, 50 μg + adjuvant group, 100 μg + adjuvant group, and 200 μg + adjuvant group, respectively. Normal saline was injected as naive control. After immunization, the serums were separated from the blood of BALB/c mice through tail vein blood collection at 0, 7, 14, 21, and 28 days. In addition, all spleens were isolated from mice at 28 days.

Supplementary Figure 2 TCID_50_ of CSFV was evaluated by indirect immunofluorescence in PK15 cells.

Supplementary Figure 3 TCID_50_ of PCV2 was evaluated by indirect immunofluorescence in PK15 cells.

Supplementary File 1 Full-length sequencing results illustrate the pSCA1-*E2-Erns-Cap-Rep* plasmid was successfully constructed.
