# Supplementary material for: Production and immunogenicity of a deoxyribonucleic acid Alphavirus vaccine expressing classical swine fever virus E2-Erns protein and porcine Circovirus Cap-Rep protein
Source: Front Microbiol. 2022 Dec 6;13:1065532. doi: 10.3389/fmicb.2022.1065532 (PMC9764008; doi:10.3389/fmicb.2022.1065532)
Supplement: Supplementary Figure 1 — Experimental design to assess the CSFV-PCV2 antibody production of pSCA1-E2-Erns-Cap-Rep plasmid in vivo. BALB/c mice were subcutaneously injected with pSCA1-E2-Erns-Cap-Rep plasmid 50 μg group, 100 μg group, 200 μg group, 50 μg + adjuvant group, 100 μg + adjuvant group, and 200 μg + adjuvant group, respectively. Normal saline was injected as naive control. After immunization, the serums were separated from the blood of BALB/c mice through tail vein blood collection at 0, 7, 14, 21, and 28 days. In addition, all spleens were isolated from mice at 28 days. [file Data_Sheet_2.pdf]

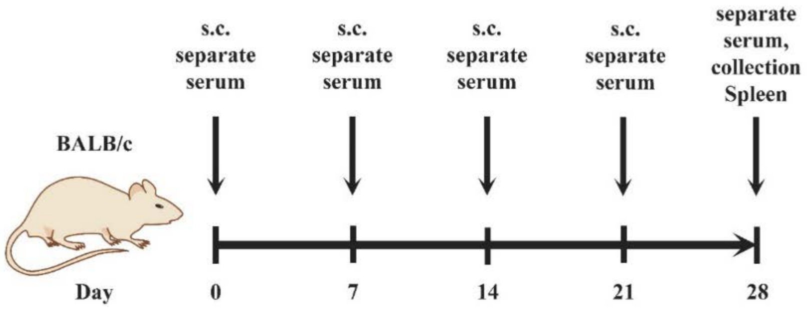

Figure S1. Experimental design to assess the CSFV-PCV2 antibody production of pSCA1-*E2-Erns-Cap-Rep* plasmid in vivo. BALB/c mice were subcutaneously injected with pSCA1-*E2-Erns-Cap-Rep* plasmid 50 µg group, 100 µg group, 200 µg group, 50 µg + adjuvant group, 100 µg + adjuvant group and 200 µg + adjuvant group, respectively. Normal saline was injected as naive control. After immunization, the serums were separated from the blood of BALB/c mice through tail vein blood collection at 0 d, 7 d, 14 d, 21 d and 28 d. In addition, all spleens were isolated from mouse at 28 d.

In review

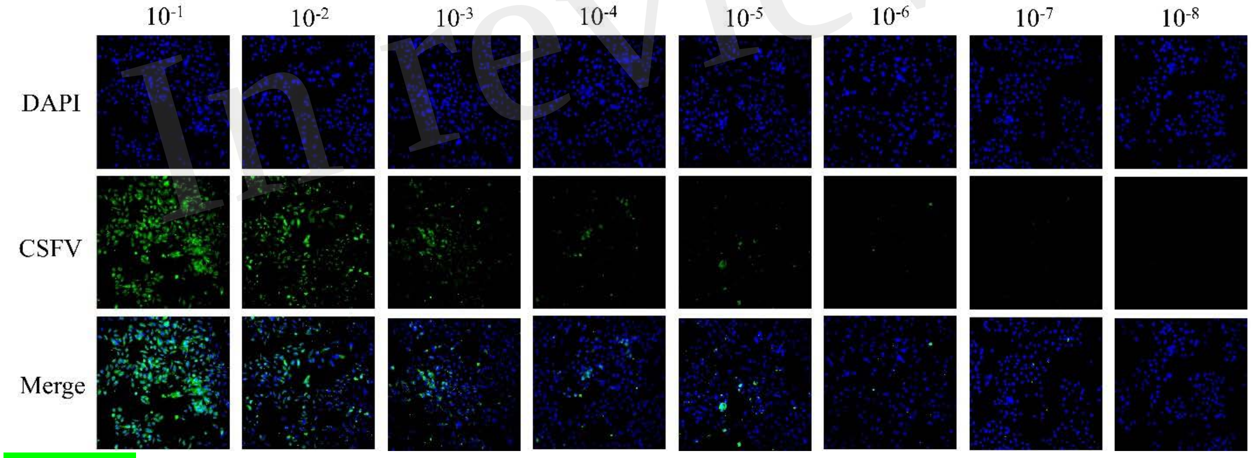

Figure S2. TCID$_{50}$ of CSFV was evaluated by indirect immunofluorescence in PK15 cells.

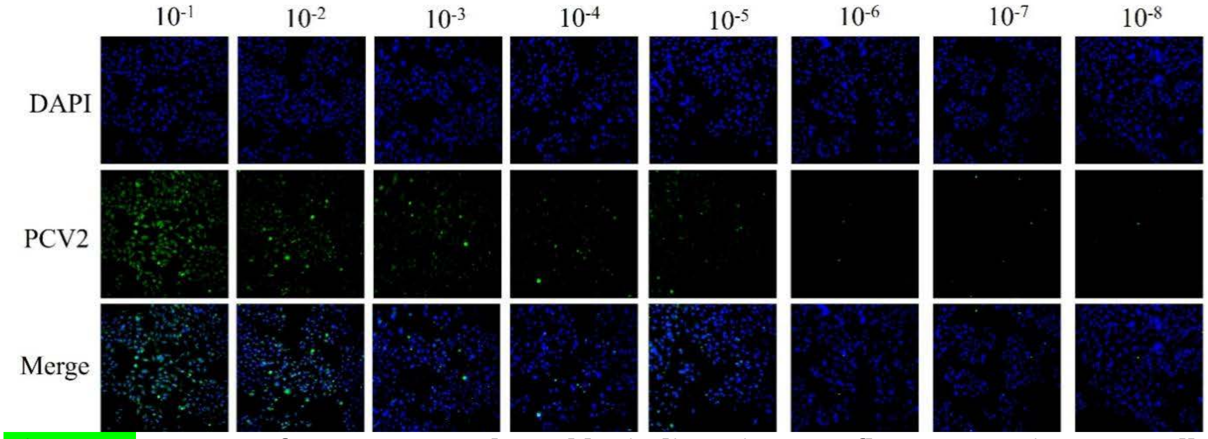

Figure S3. TCID$_{50}$ of PCV2 was evaluated by indirect immunofluorescence in PK15 cells.
