# Supplementary material for: Production and immunogenicity of a deoxyribonucleic acid Alphavirus vaccine expressing classical swine fever virus E2-Erns protein and porcine Circovirus Cap-Rep protein
Source: Front Microbiol. 2022 Dec 6;13:1065532. doi: 10.3389/fmicb.2022.1065532 (PMC9764008; doi:10.3389/fmicb.2022.1065532)
Supplement: Supplementary File 1 — Full-length sequencing results illustrate the pSCA1-E2-Erns-Cap-Rep plasmid was successfully constructed. [file Data_Sheet_1.pdf]

10 20 30 40 50 60

GGATCCATGCGGCTAGCCTGCAAGGAAGATTACAGGTACGCAATATCATCGACCAATGAGATAGGGCT

E2-P2A-Erns-P2A-Cap-P2A-Rep.seq(1>3645) → GGATCCATGCGGCTAGCCTGCAAGGAAGATTACAGGTACGCAATATCATCGACCAATGAGATAGGGCT

70 80 90 100 110 120 130

ACTCGGGGCCGAAGGTCTCACCACCACCTGGAAAGAATACAACCACGATTTGCAACTGAATGACGGGA

E2-P2A-Erns-P2A-Cap-P2A-Rep.seq(1>3645) → ACTCGGGGCCGAAGGTCTCACCACCACCTGGAAAGAATACAACCACGATTTGCAACTGAATGACGGGA

140 150 160 170 180 190 200

CCGTTAAGGCCATTTGCGTGGCAGGTTCCTTTAAAGTCATAGCACTTAATGTGGTCAGTAGGAGGTAT

E2-P2A-Erns-P2A-Cap-P2A-Rep.seq(1>3645) → CCGTTAAGGCCATTTGCGTGGCAGGTTCCTTTAAAGTCATAGCACTTAATGTGGTCAGTAGGAGGTAT

Y8847-3A(HZ275GN).1HZ275.10P_D08.scf(1>802) ←

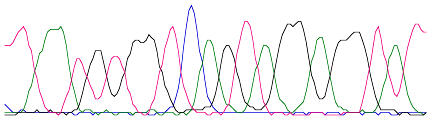

TTAATGTGGTCAGTAGGAGGTAT

210 220 230 240 250 260 270

TTGGCATCATTGCATAAGGAGGCTTCACTCACTTCCGTGACATTTGAGCTCCTGTTCGACGGGACCAA

E2-P2A-Erns-P2A-Cap-P2A-Rep.seq(1>3645) → TTGGCATCATTGCATAAGGAGGCTTCACTCACTTCCGTGACATTTGAGCTCCTGTTCGACGGGACCAA

Y8847-3A(HZ275GN).1HZ275.10P_D08.scf(1>802) ←

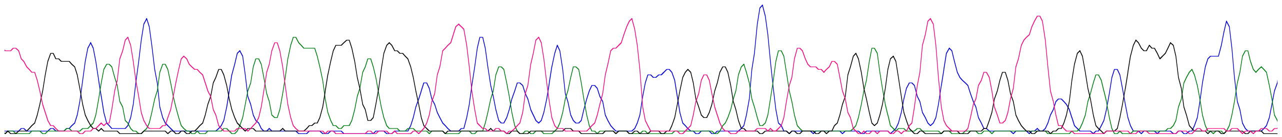

TTGGCATCATTGCATAAGGAGGCTTCACTCACTTCCGTGACATTTGAGCTCCTGTTCGACGGGACCAA

280 290 300 310 320 330 340

CCCATCAACTGAGGAAATGGGAGATGACTTCGGGTTCGGGCTGTGCCCGTTCGATACGAGTCCTGTTG

E2-P2A-Erns-P2A-Cap-P2A-Rep.seq(1>3645) → CCCATCAACTGAGGAAATGGGAGATGACTTCGGGTTCGGGCTGTGCCCGTTCGATACGAGTCCTGTTG

Y8847-3A(HZ275GN).1HZ275.10P_D08.scf(1>802) ←

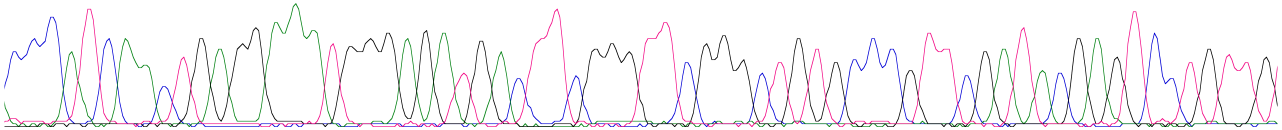

CCCATCAACTGAGGAAATGGGAGATGACTTCGGGTTCGGGCTGTGCCCGTTCGATACGAGTCCTGTTG

350 360 370 380 390 400

350 360 370 380 390 400

TCAAGGGAAAGTACAATACAACCTTGTTGAACGGTAGTGCTTTCTATCTTGTCTGCCCAATAGGGTGG

E2-P2A-Erns-P2A-Cap-P2A-Rep.seq(1>3645) → TCAAGGGAAAGTACAATACAACCTTGTTGAACGGTAGTGCTTTCTATCTTGTCTGCCCAATAGGGTGG

Y8847-3A(HZ275GN).1HZ275.10P_D08.scf(1>802) ←

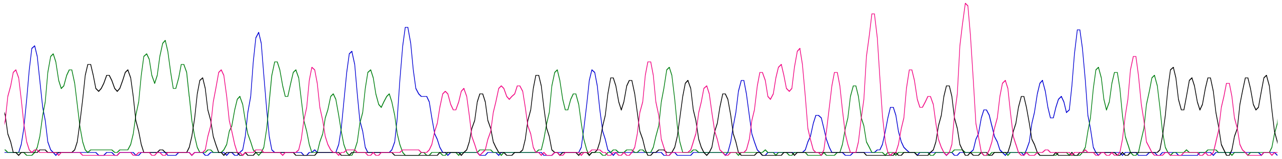

TCAAGGGAAAGTACAATACAACCTTGTTGAACGGTAGTGCTTTCTATCTTGTCTGCCCAATAGGGTGG

410 420 430 440 450 460 470

ACGGGTGTCATAGAGTGCACAGCAGTGAGCCCAACAACTCTGAGAACAGAAGTGGTAAAGACCTTCAG

E2-P2A-Erns-P2A-Cap-P2A-Rep.seq(1>3645) → ACGGGTGTCATAGAGTGCACAGCAGTGAGCCCAACAACTCTGAGAACAGAAGTGGTAAAGACCTTCAG

Y8847-3A(HZ275GN).1HZ275.10P_D08.scf(1>802) ←

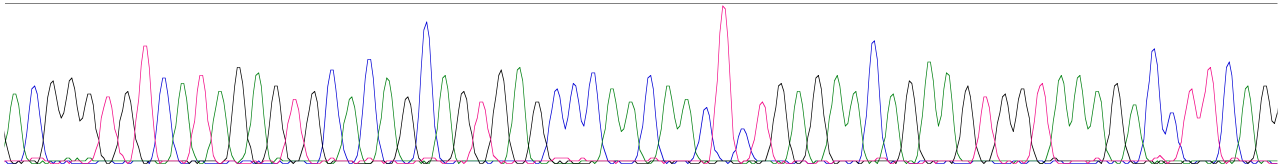

ACGGGTGTCATAGAGTGCACAGCAGTGAGCCCAACAACTCTGAGAACAGAAGTGGTAAAGACCTTCAG

480 490 500 510 520 530 540

GAGAGACAAGCCCTTTCCGCACAGAATGGATTGTGCGACCACCACAGTGGAAAATGGAGATTTATTCT

E2-P2A-Erns-P2A-Cap-P2A-Rep.seq(1>3645) → GAGAGACAAGCCCTTTCCGCACAGAATGGATTGTGCGACCACCACAGTGGAAAATGGAGATTTATTCT

Y8847-3A(HZ275GN).1HZ275.10P_D08.scf(1>802) ←

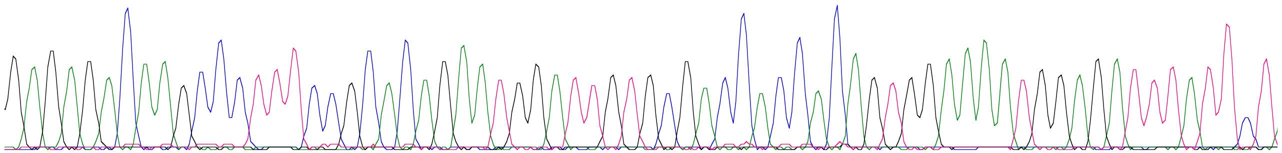

GAGAGACAAGCCCTTTCCGCACAGAATGGATTGTGCGACCACCACAGTGGAAAATGGAGATTTATTCT

Y8847-3A(HZ275GN).1HZ275.3P_E07.scf(1>785) →

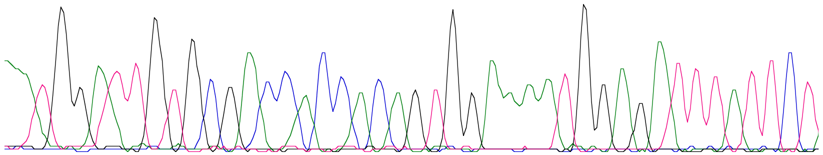

AATGGATTGTGCGACCACCACAGTGGAAAATGGAGATTTATTCT

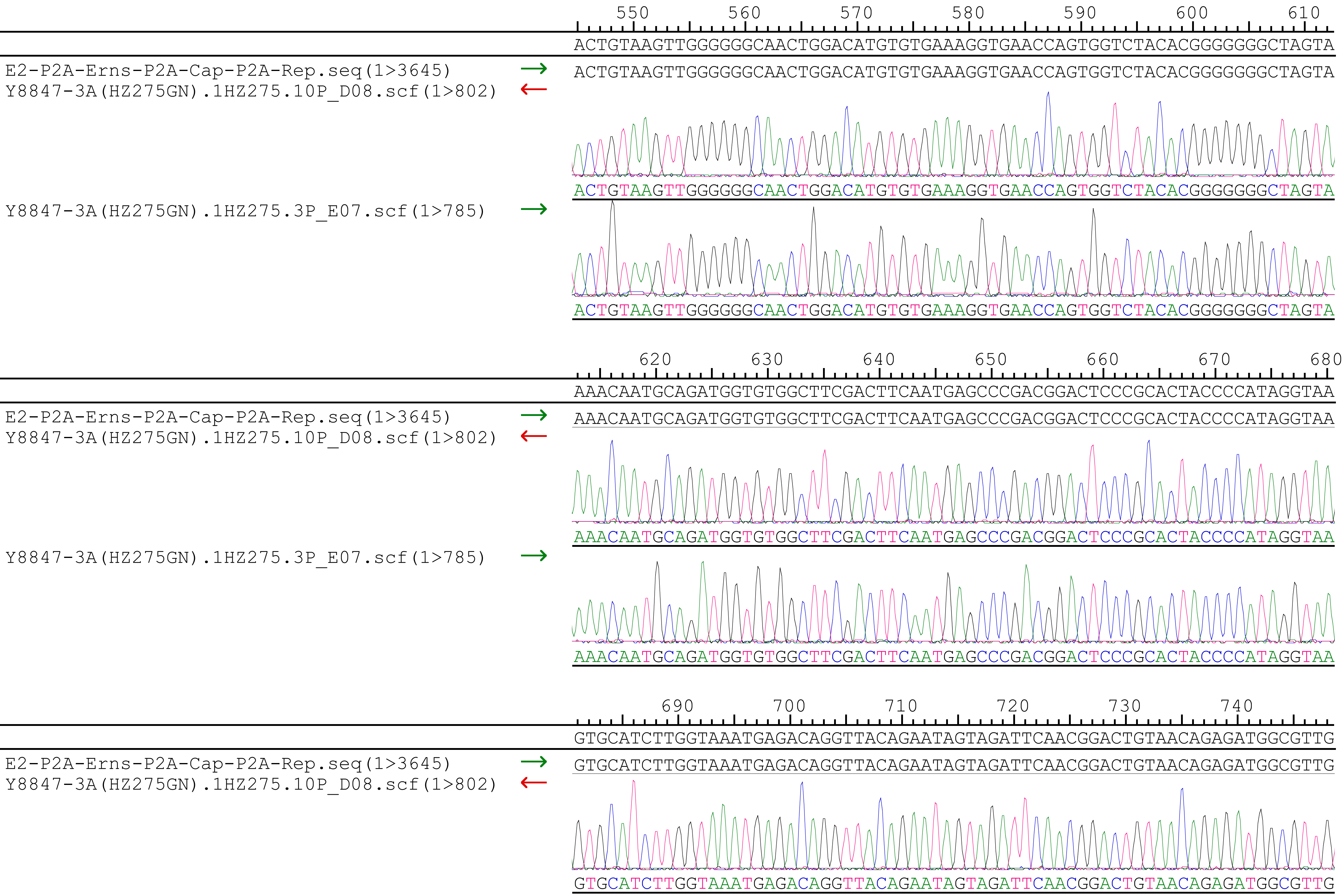
550 560 570 580 590 600 610
ACTGTAAGTTGGGGGGCAACTGGACATGTGTGAAAGGTGAACCAGTGGTCTACACGGGGGGGCTAGTA
E2-P2A-Erns-P2A-Cap-P2A-Rep.seq(1>3645)
ACTGTAAGTTGGGGGGCAACTGGACATGTGTGAAAGGTGAACCAGTGGTCTACACGGGGGGGCTAGTA
Y8847-3A(HZ275GN).1HZ275.10P_D08.scf(1>802)
ACTGTAAGTTGGGGGGCAACTGGACATGTGTGAAAGGTGAACCAGTGGTCTACACGGGGGGGCTAGTA
Y8847-3A(HZ275GN).1HZ275.3P_E07.scf(1>785)
ACTGTAAGTTGGGGGGCAACTGGACATGTGTGAAAGGTGAACCAGTGGTCTACACGGGGGGGCTAGTA
620 630 640 650 660 670 680
AAACAATGCAGATGGTGTGGCTTCGACTTCAATGAGCCCGACGGACTCCCGCACTACCCCATAGGTAA
E2-P2A-Erns-P2A-Cap-P2A-Rep.seq(1>3645)
AAACAATGCAGATGGTGTGGCTTCGACTTCAATGAGCCCGACGGACTCCCGCACTACCCCATAGGTAA
Y8847-3A(HZ275GN).1HZ275.10P_D08.scf(1>802)
AAACAATGCAGATGGTGTGGCTTCGACTTCAATGAGCCCGACGGACTCCCGCACTACCCCATAGGTAA
Y8847-3A(HZ275GN).1HZ275.3P_E07.scf(1>785)
AAACAATGCAGATGGTGTGGCTTCGACTTCAATGAGCCCGACGGACTCCCGCACTACCCCATAGGTAA
690 700 710 720 730 740
GTGCATCTTGGTAAATGAGACAGGTTACAGAATAGTAGATTCAACGGACTGTAACAGAGATGGCGTTG
E2-P2A-Erns-P2A-Cap-P2A-Rep.seq(1>3645)
GTGCATCTTGGTAAATGAGACAGGTTACAGAATAGTAGATTCAACGGACTGTAACAGAGATGGCGTTG
Y8847-3A(HZ275GN).1HZ275.10P_D08.scf(1>802)
GTGCATCTTGGTAAATGAGACAGGTTACAGAATAGTAGATTCAACGGACTGTAACAGAGATGGCGTTG

690 700 710 720 730 740

GTGCATCTTGGTAAATGAGACAGGTTACAGAATAGTAGATTCAACGGACTGTAACAGAGATGGCGTTG

Y8847-3A(HZ275GN).1HZ275.3P_E07.scf(1>785) →

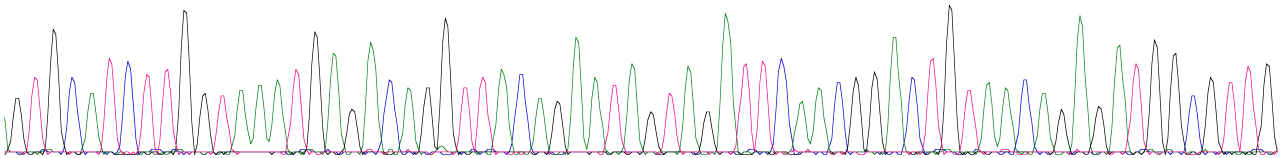

GTGCATCTTGGTAAATGAGACAGGTTACAGAATAGTAGATTCAACGGACTGTAACAGAGATGGCGTTG

750 760 770 780 790 800 810

TAATCAGCACAGATGGGAGTCATGAGTGCTTGATCGGTAACACAACTGTCAAGGTGCATGCATCAGAT

E2-P2A-Erns-P2A-Cap-P2A-Rep.seq(1>3645) → TAATCAGCACAGATGGGAGTCATGAGTGCTTGATCGGTAACACAACTGTCAAGGTGCATGCATCAGAT

Y8847-3A(HZ275GN).1HZ275.10P_D08.scf(1>802) ←

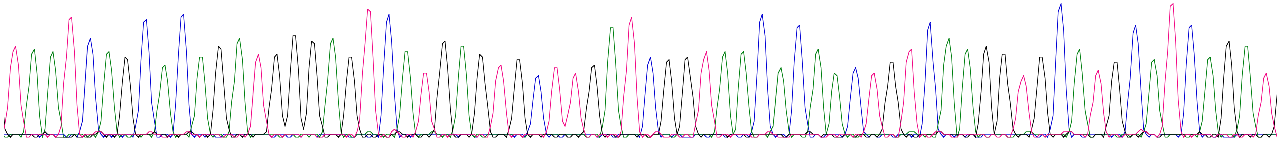

TAATCAGCACAGATGGGAGTCATGAGTGCTTGATCGGTAACACAACTGTCAAGGTGCATGCATCAGAT

Y8847-3A(HZ275GN).1HZ275.3P_E07.scf(1>785) →

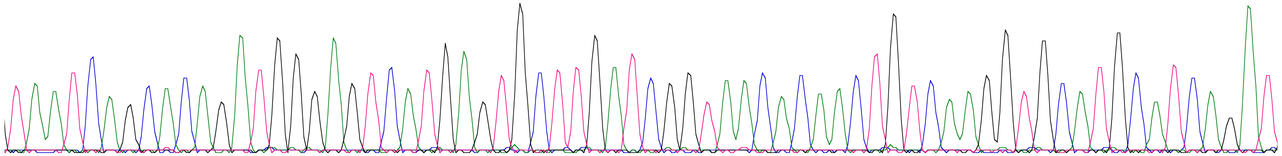

TAATCAGCACAGATGGGAGTCATGAGTGCTTGATCGGTAACACAACTGTCAAGGTGCATGCATCAGAT

820 830 840 850 860 870 880

GAAAGACTGGGCCCTATGCCATGCAGACCCAAAGAGATTGTCTCTAGTGCAGGACCTGTAAGGAAAAC

E2-P2A-Erns-P2A-Cap-P2A-Rep.seq(1>3645) → GAAAGACTGGGCCCTATGCCATGCAGACCCAAAGAGATTGTCTCTAGTGCAGGACCTGTAAGGAAAAC

Y8847-3A(HZ275GN).1HZ275.10P_D08.scf(1>802) ←

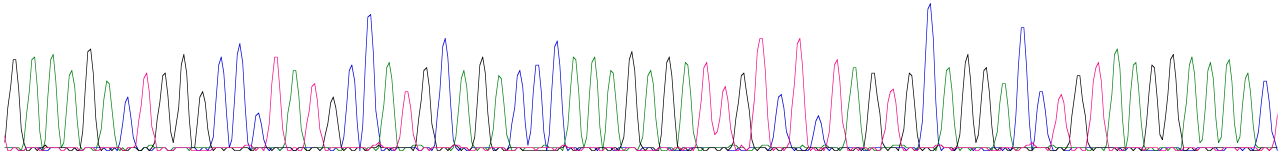

GAAAGACTGGGCCCTATGCCATGCAGACCCAAAGAGATTGTCTCTAGTGCAGGACCTGTAAGGAAAAC

Y8847-3A(HZ275GN).1HZ275.3P_E07.scf(1>785) →

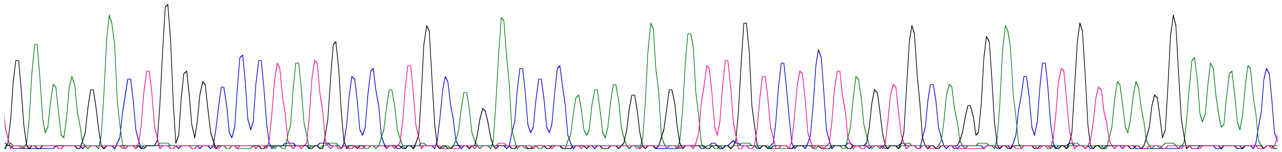

GAAAGACTGGGCCCTATGCCATGCAGACCCAAAGAGATTGTCTCTAGTGCAGGACCTGTAAGGAAAAC

890 900 910 920 930 940 950

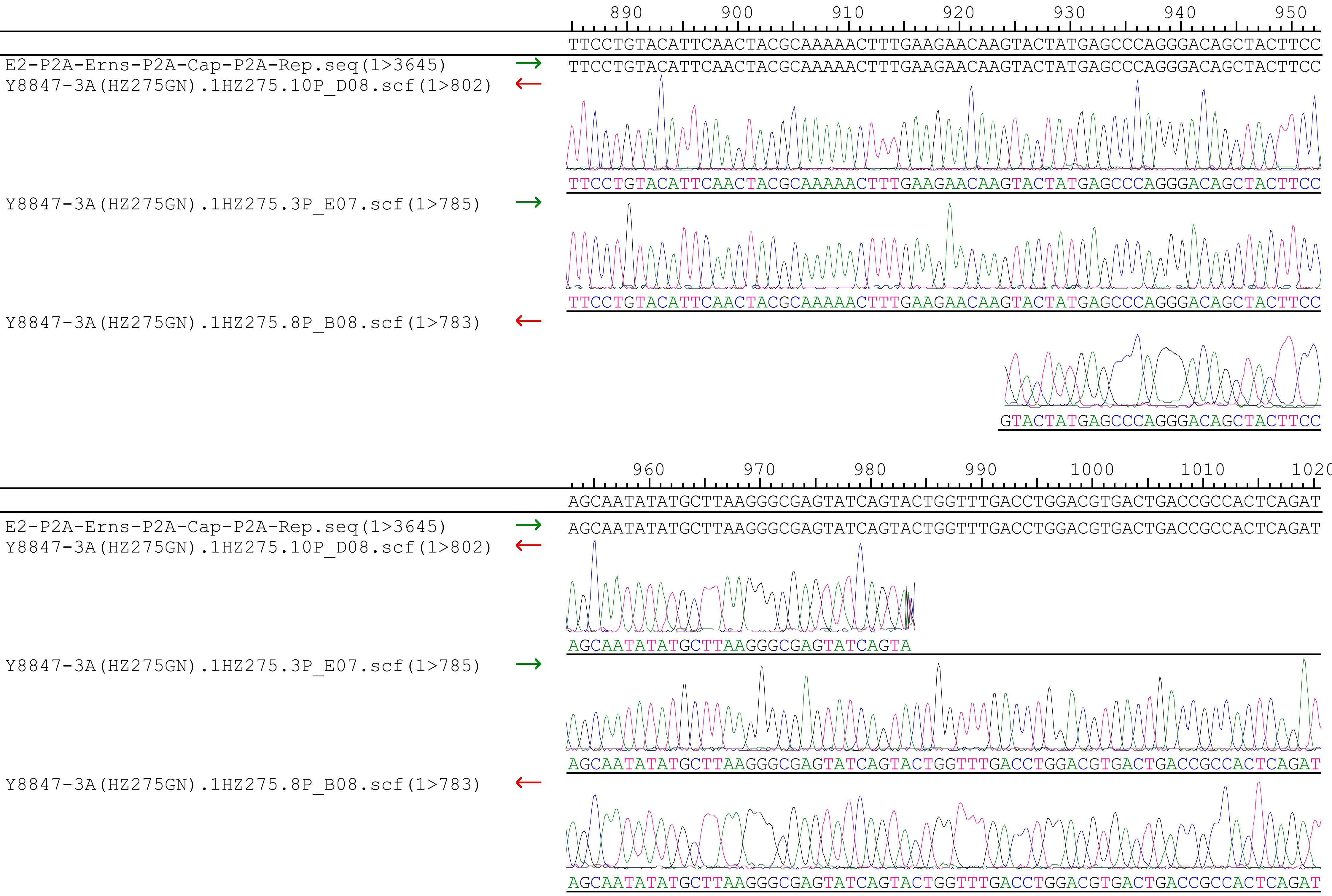
890 900 910 920 930 940 950
TTCCTGTACATTCAACTACGCAAAAACTTTGAAGAACAAGTACTATGAGCCCAGGGACAGCTACTTCC
E2-P2A-Erns-P2A-Cap-P2A-Rep.seq(1>3645)
TTCCTGTACATTCAACTACGCAAAAACTTTGAAGAACAAGTACTATGAGCCCAGGGACAGCTACTTCC
Y8847-3A(HZ275GN).1HZ275.10P_D08.scf(1>802)
TTCCTGTACATTCAACTACGCAAAAACTTTGAAGAACAAGTACTATGAGCCCAGGGACAGCTACTTCC
Y8847-3A(HZ275GN).1HZ275.3P_E07.scf(1>785)
TTCCTGTACATTCAACTACGCAAAAACTTTGAAGAACAAGTACTATGAGCCCAGGGACAGCTACTTCC
Y8847-3A(HZ275GN).1HZ275.8P_B08.scf(1>783)
GTACTATGAGCCCAGGGACAGCTACTTCC
960 970 980 990 1000 1010 1020
AGCAATATATGCTTAAGGGCGAGTATCAGTACTGGTTTGACCTGGACGTGACTGACCGCCACTCAGAT
E2-P2A-Erns-P2A-Cap-P2A-Rep.seq(1>3645)
AGCAATATATGCTTAAGGGCGAGTATCAGTACTGGTTTGACCTGGACGTGACTGACCGCCACTCAGAT
Y8847-3A(HZ275GN).1HZ275.10P_D08.scf(1>802)
AGCAATATATGCTTAAGGGCGAGTATCAGTA
Y8847-3A(HZ275GN).1HZ275.3P_E07.scf(1>785)
AGCAATATATGCTTAAGGGCGAGTATCAGTACTGGTTTGACCTGGACGTGACTGACCGCCACTCAGAT
Y8847-3A(HZ275GN).1HZ275.8P_B08.scf(1>783)
AGCAATATATGCTTAAGGGCGAGTATCAGTACTGGTTTGACCTGGACGTGACTGACCGCCACTCAGAT

960 970 980 990 1000 1010 1020

AGCAATATATGCTTAAGGGCGAGTATCAGTACTGGTTTGACCTGGACGTGACTGACCGCCACTCAGAT

Y8847-3A(HZ275GN).1HZ275.5P_G07.scf(1>763) →

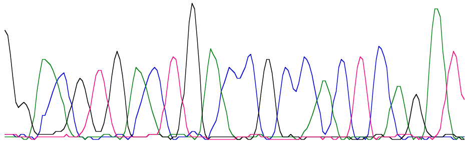

GGACGTGACTGACCGCCACTCAGAT

1030 1040 1050 1060 1070 1080

TACTTCGCAGAATTTGTCGTCTTGGTAGTGGTAGCACTGTTAGGAGGAAGATATGTCCTGTGGCTAAT

E2-P2A-Erns-P2A-Cap-P2A-Rep.seq(1>3645) → TACTTCGCAGAATTTGTCGTCTTGGTAGTGGTAGCACTGTTAGGAGGAAGATATGTCCTGTGGCTAAT

Y8847-3A(HZ275GN).1HZ275.3P_E07.scf(1>785) →

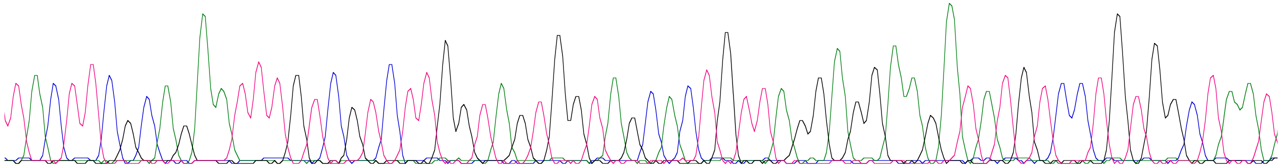

TACTTCGCAGAATTTGTCGTCTTGGTAGTGGTAGCACTGTTAGGAGGAAGATATGTCCTGTGGCTAAT

Y8847-3A(HZ275GN).1HZ275.8P_B08.scf(1>783) ←

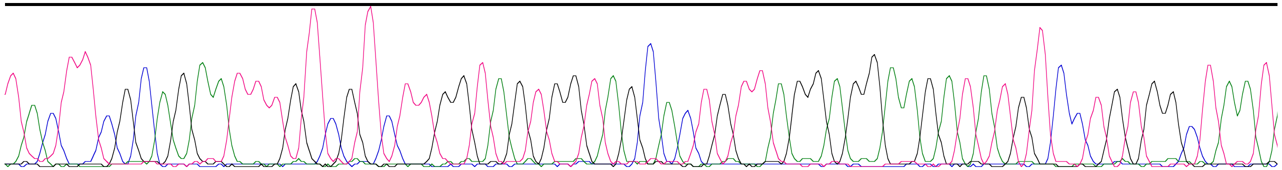

TACTTCGCAGAATTTGTCGTCTTGGTAGTGGTAGCACTGTTAGGAGGAAGATATGTCCTGTGGCTAAT

Y8847-3A(HZ275GN).1HZ275.5P_G07.scf(1>763) →

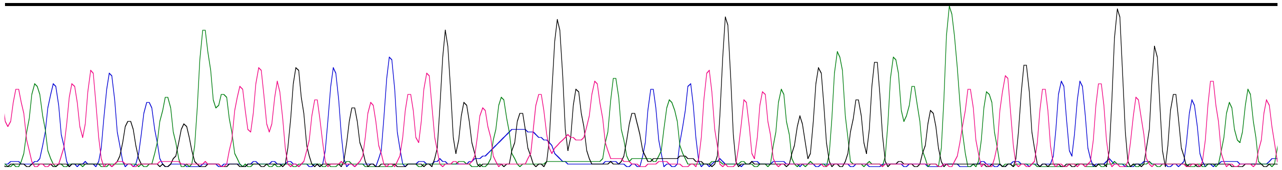

TACTTCGCAGAATTTGTCGTCTTGGTAGTGGTAGCACTGTTAGGAGGAAGATATGTCCTGTGGCTAAT

1090 1100 1110 1120 1130 1140 1150

AGTGACCTACATAGTTCTAACAGAACAACTCGCCGCTGGTGGAAGCGGAGCTACTAACTTCAGCCTGC

E2-P2A-Erns-P2A-Cap-P2A-Rep.seq(1>3645) → AGTGACCTACATAGTTCTAACAGAACAACTCGCCGCTGGTGGAAGCGGAGCTACTAACTTCAGCCTGC

Y8847-3A(HZ275GN).1HZ275.3P_E07.scf(1>785) →

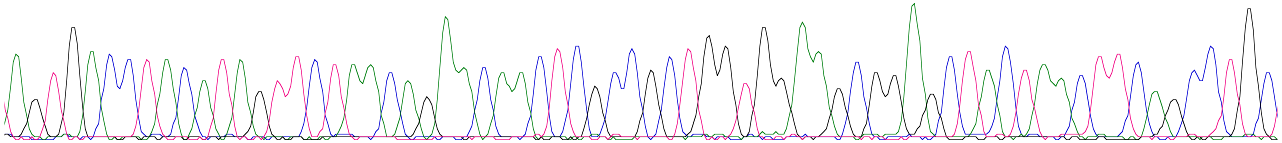

AGTGACCTACATAGTTCTAACAGAACAACTCGCCGCTGGTGGAAGCGGAGCTACTAACTTCAGCCTGC

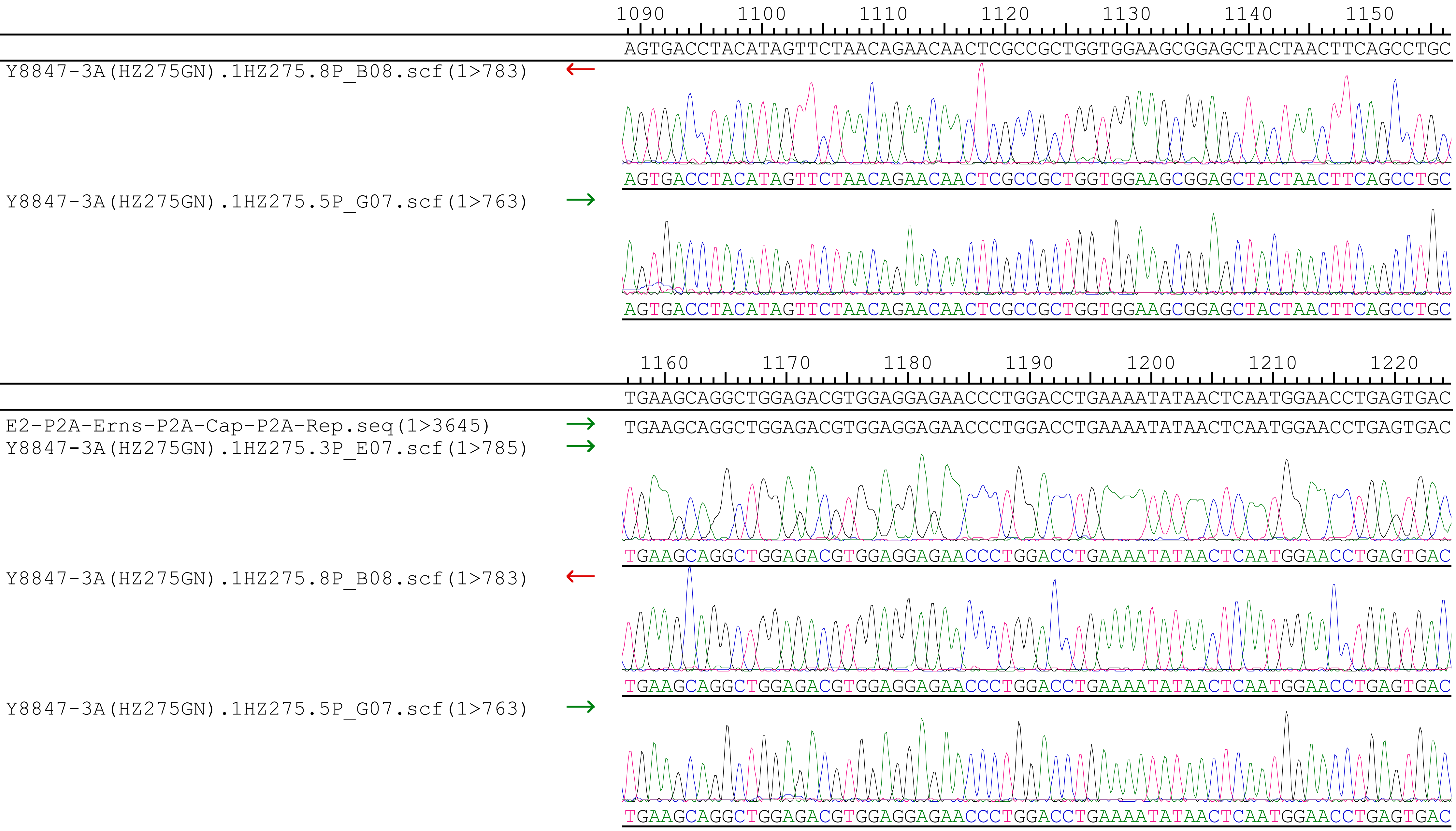

1090 1100 1110 1120 1130 1140 1150
AGTGACCTACATAGTTCTAACAGAACAACTCGCCGCTGGTGGAAGCGGAGCTACTAACTTCAGCCTGC
Y8847-3A(HZ275GN).1HZ275.8P_B08.scf(1>783)
AGTGACCTACATAGTTCTAACAGAACAACTCGCCGCTGGTGGAAGCGGAGCTACTAACTTCAGCCTGC
Y8847-3A(HZ275GN).1HZ275.5P_G07.scf(1>763)
AGTGACCTACATAGTTCTAACAGAACAACTCGCCGCTGGTGGAAGCGGAGCTACTAACTTCAGCCTGC
1160 1170 1180 1190 1200 1210 1220
TGAAGCAGGCTGGAGACGTGGAGGAGAACCCTGGACCTGAAAATATAACTCAATGGAACCTGAGTGAC
E2-P2A-Erns-P2A-Cap-P2A-Rep.seq(1>3645)
TGAAGCAGGCTGGAGACGTGGAGGAGAACCCTGGACCTGAAAATATAACTCAATGGAACCTGAGTGAC
Y8847-3A(HZ275GN).1HZ275.3P_E07.scf(1>785)
TGAAGCAGGCTGGAGACGTGGAGGAGAACCCTGGACCTGAAAATATAACTCAATGGAACCTGAGTGAC
Y8847-3A(HZ275GN).1HZ275.8P_B08.scf(1>783)
TGAAGCAGGCTGGAGACGTGGAGGAGAACCCTGGACCTGAAAATATAACTCAATGGAACCTGAGTGAC
Y8847-3A(HZ275GN).1HZ275.5P_G07.scf(1>763)
TGAAGCAGGCTGGAGACGTGGAGGAGAACCCTGGACCTGAAAATATAACTCAATGGAACCTGAGTGAC

1230 1240 1250 1260 1270 1280 1290

AACGGCACTAATGGTATCCAGCATGCTATGTACCTTAGAGGGGTTAGCAGGAGCTTGCATGGGATCTG

E2-P2A-Erns-P2A-Cap-P2A-Rep.seq(1>3645) → AACGGCACTAATGGTATCCAGCATGCTATGTACCTTAGAGGGGTTAGCAGGAGCTTGCATGGGATCTG

Y8847-3A(HZ275GN).1HZ275.3P_E07.scf(1>785) →

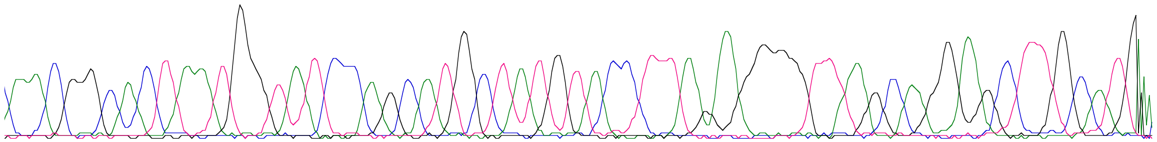

AACGGCACTAATGGTATCCAGCATGCTATGTACCTTAGAGGGGTTAGCAGGAGCTTGCATG

Y8847-3A(HZ275GN).1HZ275.8P_B08.scf(1>783) ←

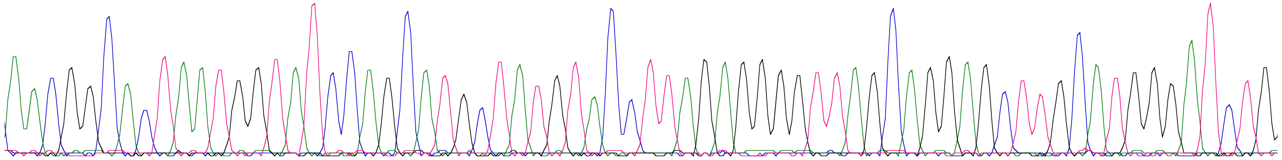

AACGGCACTAATGGTATCCAGCATGCTATGTACCTTAGAGGGGTTAGCAGGAGCTTGCATGGGATCTG

Y8847-3A(HZ275GN).1HZ275.5P_G07.scf(1>763) →

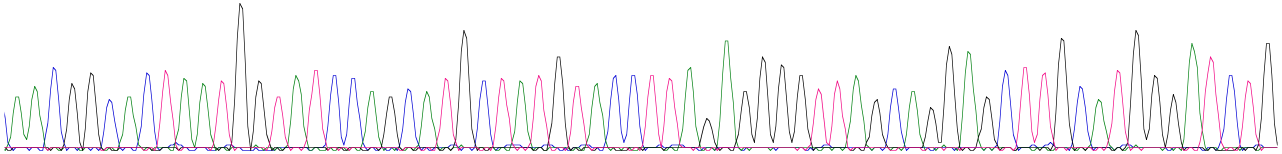

AACGGCACTAATGGTATCCAGCATGCTATGTACCTTAGAGGGGTTAGCAGGAGCTTGCATGGGATCTG

1300 1310 1320 1330 1340 1350 1360

GCCGGAAAAAATATGCAAAGGAGTCCCCACCTACCTGGCCACAGACACGGAACTGAAAGAAATACAGG

E2-P2A-Erns-P2A-Cap-P2A-Rep.seq(1>3645) → GCCGGAAAAAATATGCAAAGGAGTCCCCACCTACCTGGCCACAGACACGGAACTGAAAGAAATACAGG

Y8847-3A(HZ275GN).1HZ275.8P_B08.scf(1>783) ←

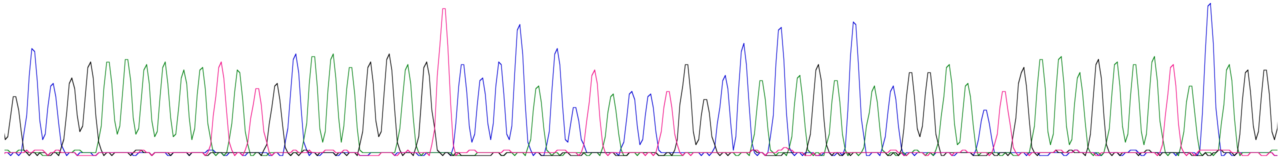

GCCGGAAAAAATATGCAAAGGAGTCCCCACCTACCTGGCCACAGACACGGAACTGAAAGAAATACAGG

Y8847-3A(HZ275GN).1HZ275.5P_G07.scf(1>763) →

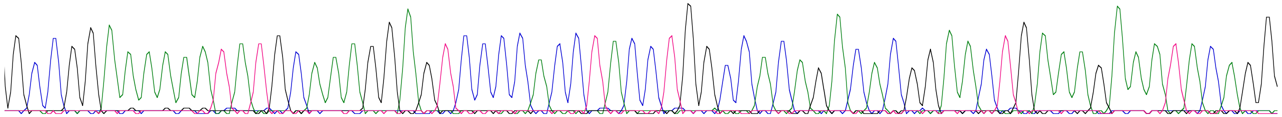

GCCGGAAAAAATATGCAAAGGAGTCCCCACCTACCTGGCCACAGACACGGAACTGAAAGAAATACAGG

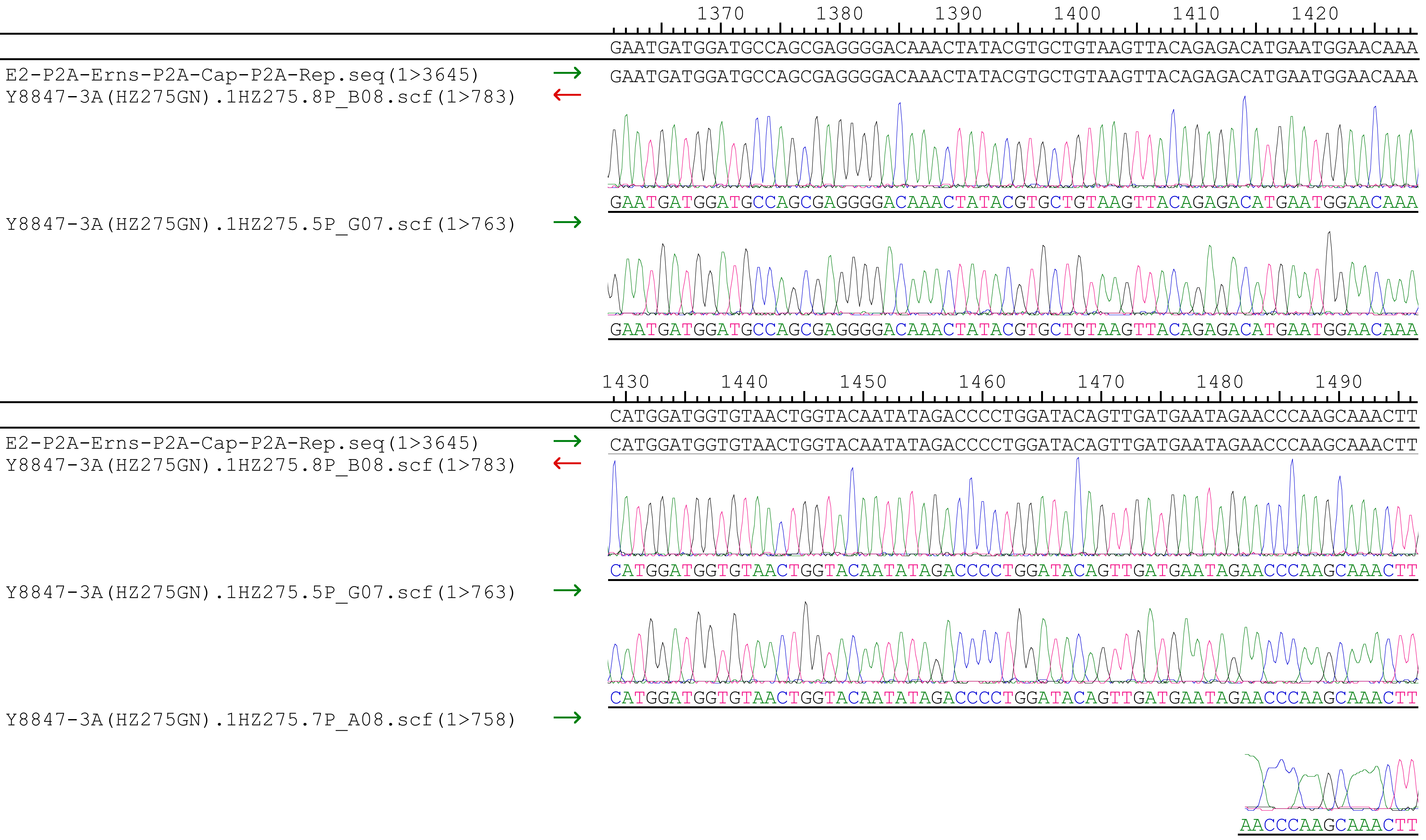
1370 1380 1390 1400 1410 1420
GAATGATGGATGCCAGCGAGGGGACAAACTATACGTGCTGTAAGTTACAGAGACATGAATGGAACAAA
E2-P2A-Erns-P2A-Cap-P2A-Rep.seq(1>3645)
GAATGATGGATGCCAGCGAGGGGACAAACTATACGTGCTGTAAGTTACAGAGACATGAATGGAACAAA
Y8847-3A(HZ275GN).1HZ275.8P_B08.scf(1>783)
GAATGATGGATGCCAGCGAGGGGACAAACTATACGTGCTGTAAGTTACAGAGACATGAATGGAACAAA
Y8847-3A(HZ275GN).1HZ275.5P_G07.scf(1>763)
GAATGATGGATGCCAGCGAGGGGACAAACTATACGTGCTGTAAGTTACAGAGACATGAATGGAACAAA
1430 1440 1450 1460 1470 1480 1490
CATGGATGGTGTAACTGGTACAATATAGACCCCTGGATACAGTTGATGAATAGAACCCAAGCAAACTT
E2-P2A-Erns-P2A-Cap-P2A-Rep.seq(1>3645)
CATGGATGGTGTAACTGGTACAATATAGACCCCTGGATACAGTTGATGAATAGAACCCAAGCAAACTT
Y8847-3A(HZ275GN).1HZ275.8P_B08.scf(1>783)
CATGGATGGTGTAACTGGTACAATATAGACCCCTGGATACAGTTGATGAATAGAACCCAAGCAAACTT
Y8847-3A(HZ275GN).1HZ275.5P_G07.scf(1>763)
CATGGATGGTGTAACTGGTACAATATAGACCCCTGGATACAGTTGATGAATAGAACCCAAGCAAACTT
Y8847-3A(HZ275GN).1HZ275.7P_A08.scf(1>758)
AACCCAAGCAAACTT

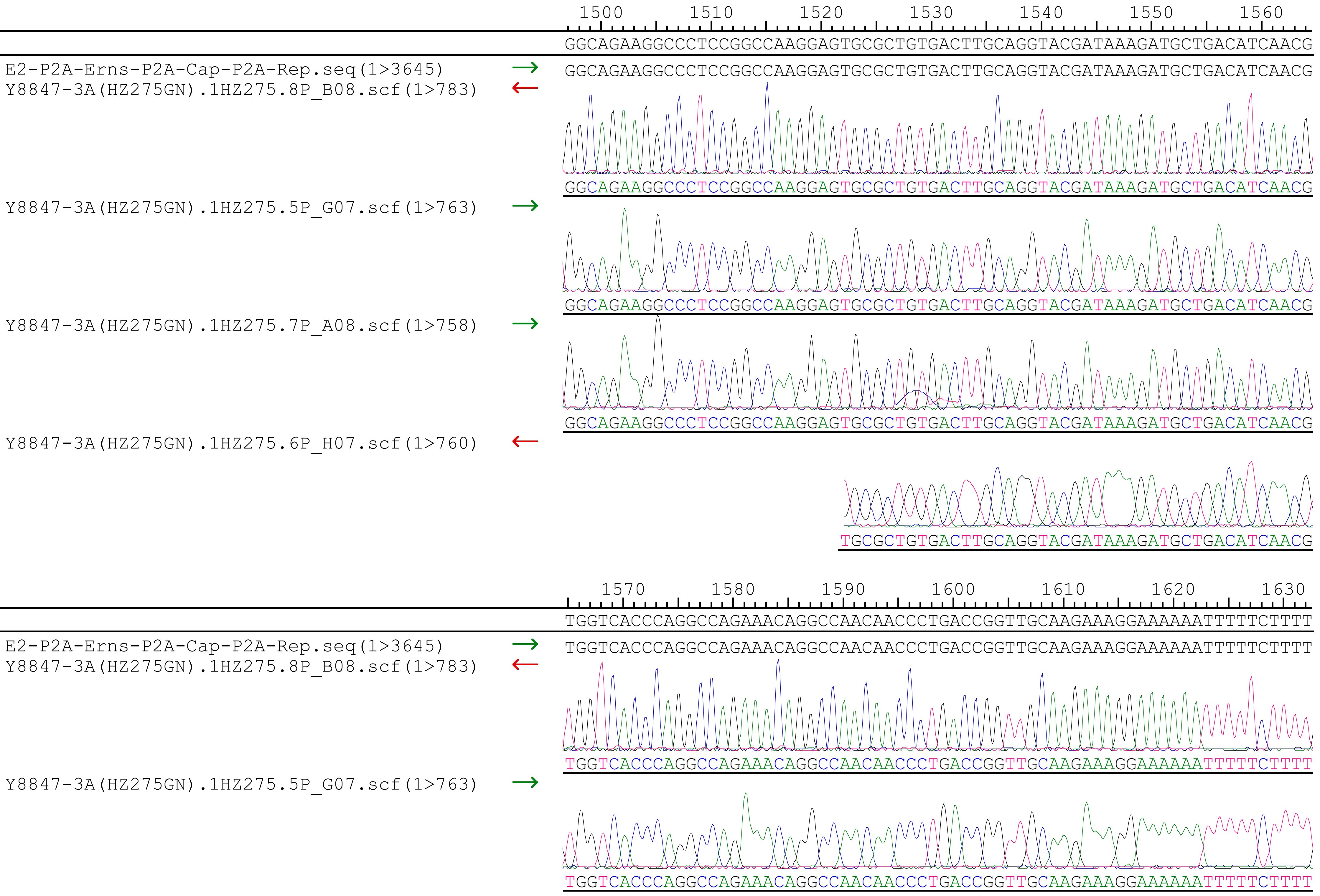
1500 1510 1520 1530 1540 1550 1560
GGCAGAAGGCCCTCCGGCCAAGGAGTGCGCTGTGACTTGCAGGTACGATAAAGATGCTGACATCAACG
E2-P2A-Erns-P2A-Cap-P2A-Rep.seq(1>3645)
GGCAGAAGGCCCTCCGGCCAAGGAGTGCGCTGTGACTTGCAGGTACGATAAAGATGCTGACATCAACG
Y8847-3A(HZ275GN).1HZ275.8P_B08.scf(1>783)
GGCAGAAGGCCCTCCGGCCAAGGAGTGCGCTGTGACTTGCAGGTACGATAAAGATGCTGACATCAACG
Y8847-3A(HZ275GN).1HZ275.5P_G07.scf(1>763)
GGCAGAAGGCCCTCCGGCCAAGGAGTGCGCTGTGACTTGCAGGTACGATAAAGATGCTGACATCAACG
Y8847-3A(HZ275GN).1HZ275.7P_A08.scf(1>758)
GGCAGAAGGCCCTCCGGCCAAGGAGTGCGCTGTGACTTGCAGGTACGATAAAGATGCTGACATCAACG
Y8847-3A(HZ275GN).1HZ275.6P_H07.scf(1>760)
TGCGCTGTGACTTGCAGGTACGATAAAGATGCTGACATCAACG
1570 1580 1590 1600 1610 1620 1630
TGGTCACCCAGGCCAGAAACAGGCCAACAACCCTGACCGGTTGCAAGAAAGGAAAAAATTTTTCTTTT
E2-P2A-Erns-P2A-Cap-P2A-Rep.seq(1>3645)
TGGTCACCCAGGCCAGAAACAGGCCAACAACCCTGACCGGTTGCAAGAAAGGAAAAAATTTTTCTTTT
Y8847-3A(HZ275GN).1HZ275.8P_B08.scf(1>783)
TGGTCACCCAGGCCAGAAACAGGCCAACAACCCTGACCGGTTGCAAGAAAGGAAAAAATTTTTCTTTT
Y8847-3A(HZ275GN).1HZ275.5P_G07.scf(1>763)
TGGTCACCCAGGCCAGAAACAGGCCAACAACCCTGACCGGTTGCAAGAAAGGAAAAAATTTTTCTTTT

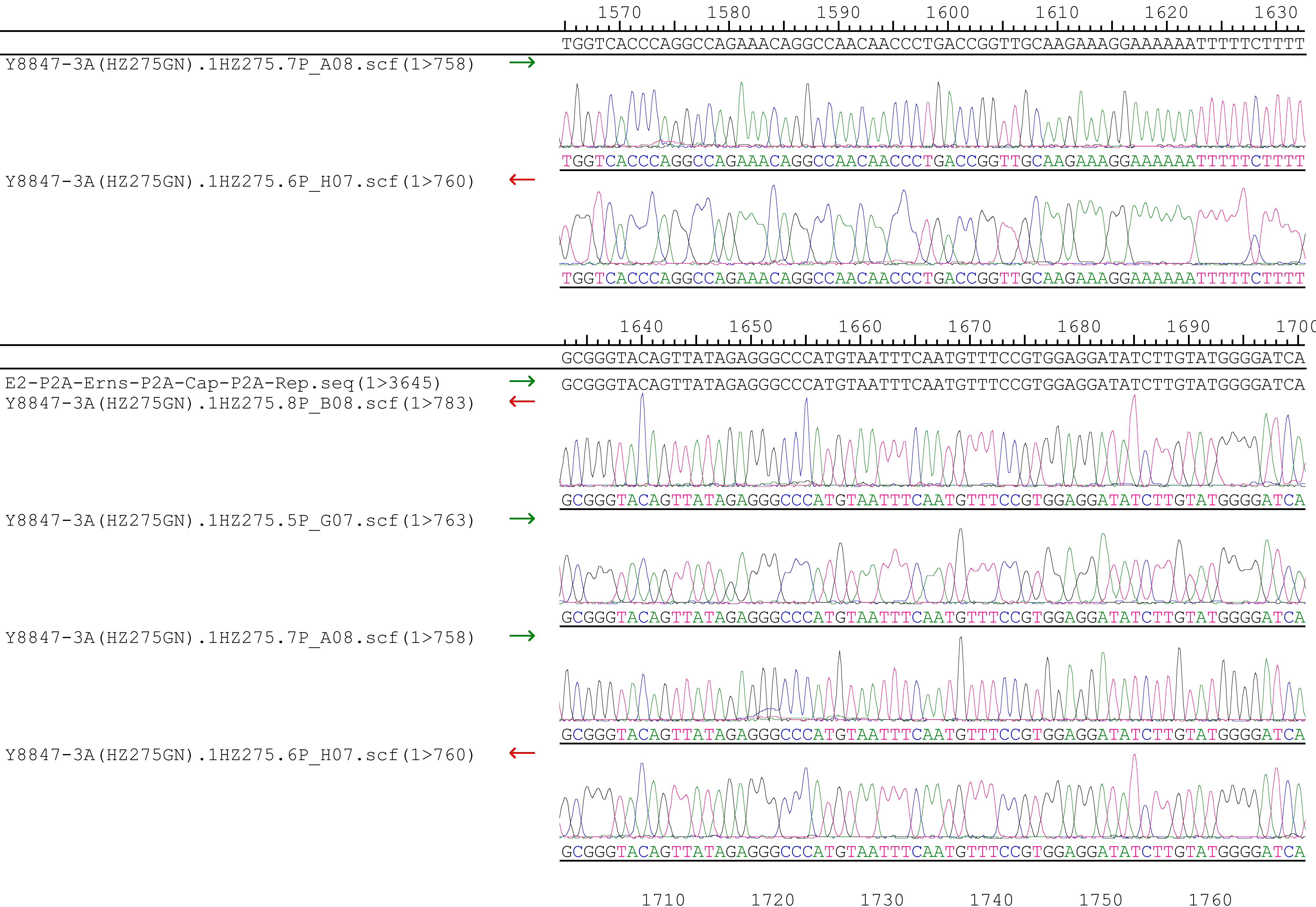
1570 1580 1590 1600 1610 1620 1630
TGGTCACCCAGGCCAGAAACAGGCCAACAACCCTGACCGGTTGCAAGAAAGGAAAAAATTTTTCTTTT
Y8847-3A(HZ275GN).1HZ275.7P_A08.scf(1>758)
TGGTCACCCAGGCCAGAAACAGGCCAACAACCCTGACCGGTTGCAAGAAAGGAAAAAATTTTTCTTTT
Y8847-3A(HZ275GN).1HZ275.6P_H07.scf(1>760)
TGGTCACCCAGGCCAGAAACAGGCCAACAACCCTGACCGGTTGCAAGAAAGGAAAAAATTTTTCTTTT
1640 1650 1660 1670 1680 1690 170
GCGGGTACAGTTATAGAGGGCCCATGTAATTTCAATGTTTCCGTGGAGGATATCTTGTATGGGGATCA
E2-P2A-Erns-P2A-Cap-P2A-Rep.seq(1>3645)
GCGGGTACAGTTATAGAGGGCCCATGTAATTTCAATGTTTCCGTGGAGGATATCTTGTATGGGGATCA
Y8847-3A(HZ275GN).1HZ275.8P_B08.scf(1>783)
GCGGGTACAGTTATAGAGGGCCCATGTAATTTCAATGTTTCCGTGGAGGATATCTTGTATGGGGATCA
Y8847-3A(HZ275GN).1HZ275.5P_G07.scf(1>763)
GCGGGTACAGTTATAGAGGGCCCATGTAATTTCAATGTTTCCGTGGAGGATATCTTGTATGGGGATCA
Y8847-3A(HZ275GN).1HZ275.7P_A08.scf(1>758)
GCGGGTACAGTTATAGAGGGCCCATGTAATTTCAATGTTTCCGTGGAGGATATCTTGTATGGGGATCA
Y8847-3A(HZ275GN).1HZ275.6P_H07.scf(1>760)
GCGGGTACAGTTATAGAGGGCCCATGTAATTTCAATGTTTCCGTGGAGGATATCTTGTATGGGGATCA
1710 1720 1730 1740 1750 1760

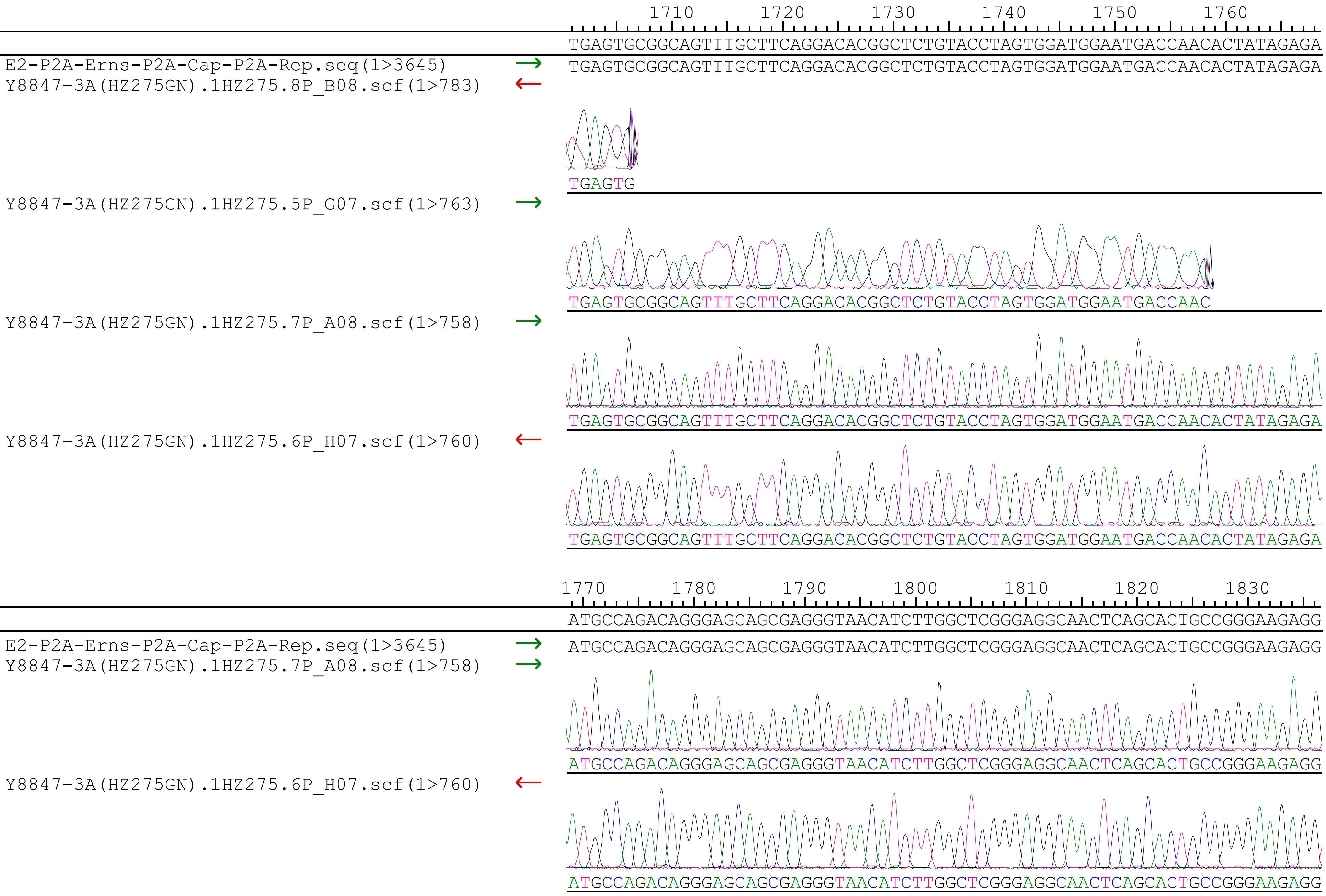
1710 1720 1730 1740 1750 1760
TGAGTGCGGCAGTTTGCTTCAGGACACGGCTCTGTACCTAGTGGATGGAATGACCAACACTATAGAGA
E2-P2A-Erns-P2A-Cap-P2A-Rep.seq(1>3645)
TGAGTGCGGCAGTTTGCTTCAGGACACGGCTCTGTACCTAGTGGATGGAATGACCAACACTATAGAGA
Y8847-3A(HZ275GN).1HZ275.8P_B08.scf(1>783)
TGAGTG
Y8847-3A(HZ275GN).1HZ275.5P_G07.scf(1>763)
TGAGTGCGGCAGTTTGCTTCAGGACACGGCTCTGTACCTAGTGGATGGAATGACCAAC
Y8847-3A(HZ275GN).1HZ275.7P_A08.scf(1>758)
TGAGTGCGGCAGTTTGCTTCAGGACACGGCTCTGTACCTAGTGGATGGAATGACCAACACTATAGAGA
Y8847-3A(HZ275GN).1HZ275.6P_H07.scf(1>760)
TGAGTGCGGCAGTTTGCTTCAGGACACGGCTCTGTACCTAGTGGATGGAATGACCAACACTATAGAGA
1770 1780 1790 1800 1810 1820 1830
ATGCCAGACAGGGAGCAGCGAGGGTAACATCTTGGCTCGGGAGGCAACTCAGCACTGCCGGGAAGAGG
E2-P2A-Erns-P2A-Cap-P2A-Rep.seq(1>3645)
ATGCCAGACAGGGAGCAGCGAGGGTAACATCTTGGCTCGGGAGGCAACTCAGCACTGCCGGGAAGAGG
Y8847-3A(HZ275GN).1HZ275.7P_A08.scf(1>758)
ATGCCAGACAGGGAGCAGCGAGGGTAACATCTTGGCTCGGGAGGCAACTCAGCACTGCCGGGAAGAGG
Y8847-3A(HZ275GN).1HZ275.6P_H07.scf(1>760)
ATGCCAGACAGGGAGCAGCGAGGGTAACATCTTGGCTCGGGAGGCAACTCAGCACTGCCGGGAAGAGG

1840 1850 1860 1870 1880 1890 1900

TTGGAGGGTAGAAGCAAAACCTGGTTTGGTGCCTATGCCGGAAGCGGAGCTACTAACTTCAGCCTGCT

E2-P2A-Erns-P2A-Cap-P2A-Rep.seq(1>3645) → TTGGAGGGTAGAAGCAAAACCTGGTTTGGTGCCTATGCCGGAAGCGGAGCTACTAACTTCAGCCTGCT

Y8847-3A(HZ275GN).1HZ275.7P_A08.scf(1>758) →

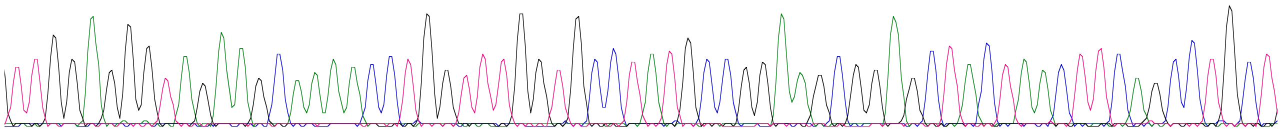

TTGGAGGGTAGAAGCAAAACCTGGTTTGGTGCCTATGCCGGAAGCGGAGCTACTAACTTCAGCCTGCT

Y8847-3A(HZ275GN).1HZ275.6P_H07.scf(1>760) ←

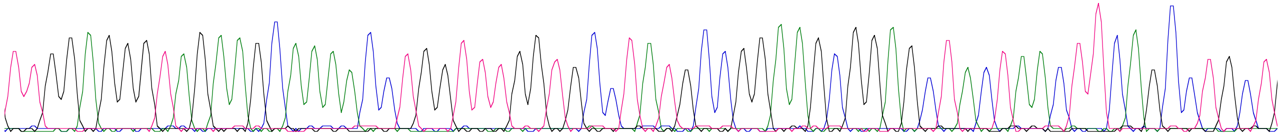

TTGGAGGGTAGAAGCAAAACCTGGTTTGGTGCCTATGCCGGAAGCGGAGCTACTAACTTCAGCCTGCT

1910 1920 1930 1940 1950 1960 1970

GAAGCAGGCTGGAGACGTGGAGGAGAACCCTGGACCTACGTATCCAAGGAGGCGTTACCGGAGAAGAA

E2-P2A-Erns-P2A-Cap-P2A-Rep.seq(1>3645) → GAAGCAGGCTGGAGACGTGGAGGAGAACCCTGGACCTACGTATCCAAGGAGGCGTTACCGGAGAAGAA

Y8847-3A(HZ275GN).1HZ275.7P_A08.scf(1>758) →

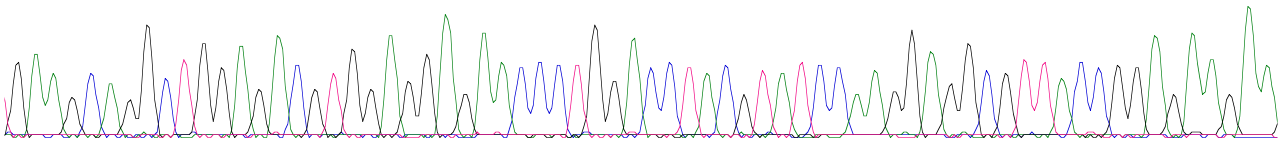

GAAGCAGGCTGGAGACGTGGAGGAGAACCCTGGACCTACGTATCCAAGGAGGCGTTACCGGAGAAGAA

Y8847-3A(HZ275GN).1HZ275.6P_H07.scf(1>760) ←

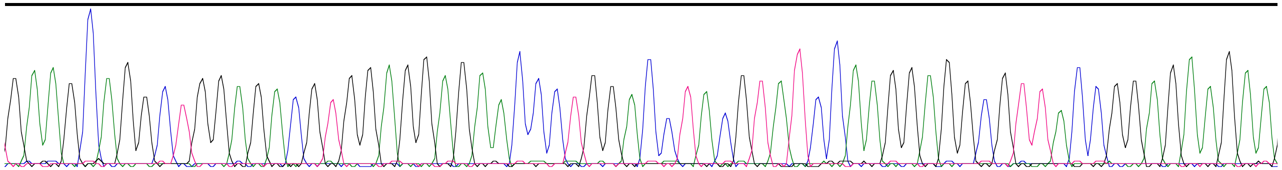

GAAGCAGGCTGGAGACGTGGAGGAGAACCCTGGACCTACGTATCCAAGGAGGCGTTACCGGAGAAGAA

1980 1990 2000 2010 2020 2030 2040

GACACCGCCCCCGCAGCCATCTTGGCCAGATCCTCCGCCGCCGCCCCTGGCTCGTCCACCCCCGCCAC

E2-P2A-Erns-P2A-Cap-P2A-Rep.seq(1>3645) → GACACCGCCCCCGCAGCCATCTTGGCCAGATCCTCCGCCGCCGCCCCTGGCTCGTCCACCCCCGCCAC

Y8847-3A(HZ275GN).1HZ275.7P_A08.scf(1>758) →

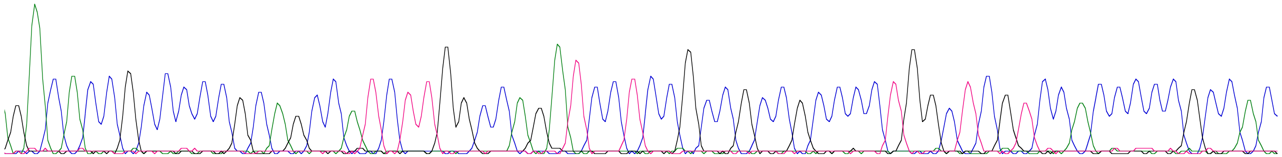

GACACCGCCCCCGCAGCCATCTTGGCCAGATCCTCCGCCGCCGCCCCTGGCTCGTCCACCCCCGCCAC

1980 1990 2000 2010 2020 2030 2040

GACACCGCCCCCGCAGCCATCTTGGCCAGATCCTCCGCCGCCGCCCCTGGCTCGTCCACCCCCGCCAC

Y8847-3A(HZ275GN).1HZ275.6P_H07.scf(1>760) ←

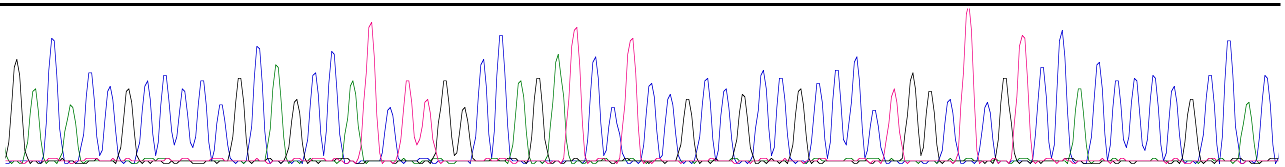

GACACCGCCCCCGCAGCCATCTTGGCCAGATCCTCCGCCGCCGCCCCTGGCTCGTCCACCCCCGCCAC

Y8847-3A(HZ275GN).1HZ275.9P_C08.scf(1>787) →

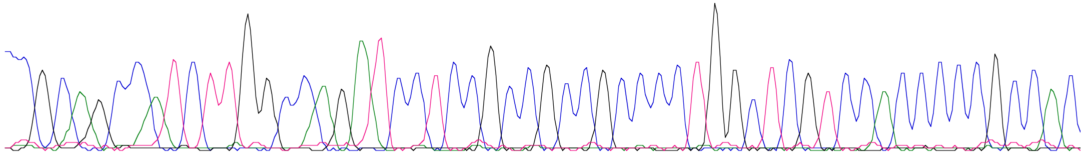

CCGCAGCCATCTTGGCCAGATCCTCCGCCGCCGCCCCTGGCTCGTCCACCCCCGCCAC

2050 2060 2070 2080 2090 2100

CGTTACCGCTGGAGAAGGAAAAATGGCATCTTCAACACCCGCCTATCCCGCACCTTCGGATATACTAT

E2-P2A-Erns-P2A-Cap-P2A-Rep.seq(1>3645) → CGTTACCGCTGGAGAAGGAAAAATGGCATCTTCAACACCCGCCTATCCCGCACCTTCGGATATACTAT

Y8847-3A(HZ275GN).1HZ275.7P_A08.scf(1>758) →

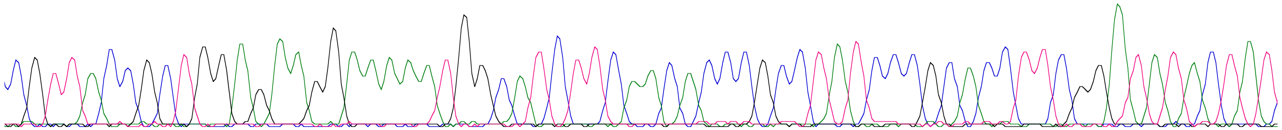

CGTTACCGCTGGAGAAGGAAAAATGGCATCTTCAACACCCGCCTATCCCGCACCTTCGGATATACTAT

Y8847-3A(HZ275GN).1HZ275.6P_H07.scf(1>760) ←

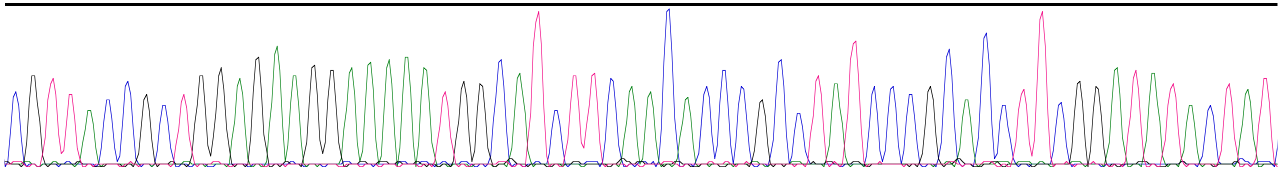

CGTTACCGCTGGAGAAGGAAAAATGGCATCTTCAACACCCGCCTATCCCGCACCTTCGGATATACTAT

Y8847-3A(HZ275GN).1HZ275.9P_C08.scf(1>787) →

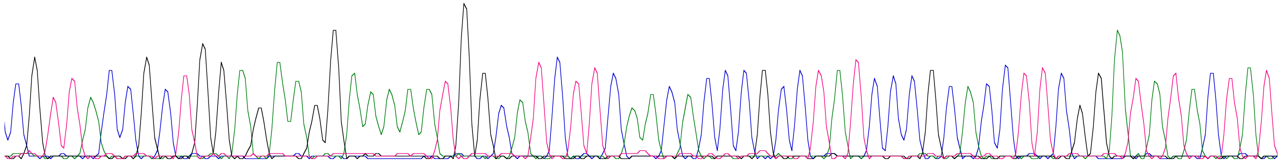

CGTTACCGCTGGAGAAGGAAAAATGGCATCTTCAACACCCGCCTATCCCGCACCTTCGGATATACTAT

2110 2120 2130 2140 2150 2160 2170

CAAGCGAACCACAGTCAGAACGCCCTCCTGGGCGGTGGACATGATGAGATTCAATATTAATGACTTTC

E2-P2A-Erns-P2A-Cap-P2A-Rep.seq(1>3645) → CAAGCGAACCACAGTCAGAACGCCCTCCTGGGCGGTGGACATGATGAGATTCAATATTAATGACTTTC

Y8847-3A(HZ275GN).1HZ275.7P_A08.scf(1>758) →

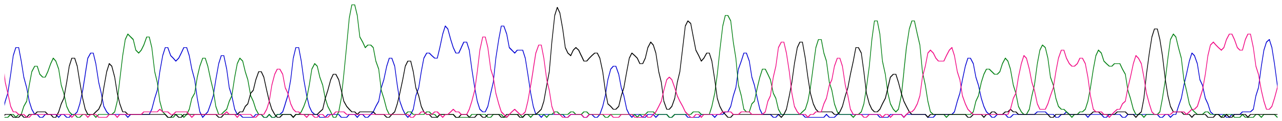

CAAGCGAACCACAGTCAGAACGCCCTCCTGGGCGGTGGACATGATGAGATTCAATATTAATGACTTTC

Y8847-3A(HZ275GN).1HZ275.6P_H07.scf(1>760) ←

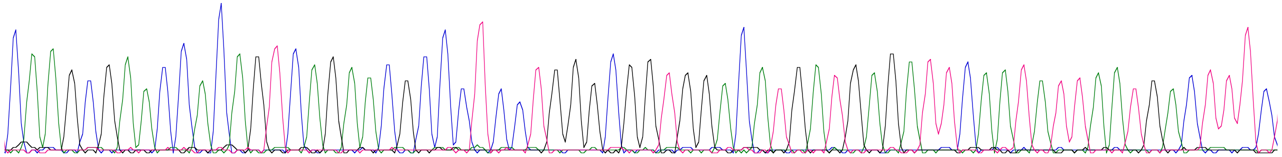

CAAGCGAACCACAGTCAGAACGCCCTCCTGGGCGGTGGACATGATGAGATTCAATATTAATGACTTTC

Y8847-3A(HZ275GN).1HZ275.9P_C08.scf(1>787) →

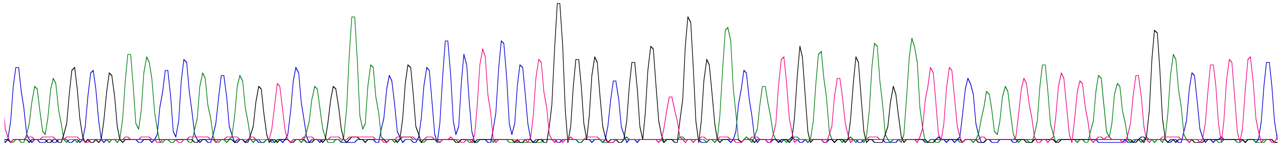

CAAGCGAACCACAGTCAGAACGCCCTCCTGGGCGGTGGACATGATGAGATTCAATATTAATGACTTTC

2180 2190 2200 2210 2220 2230 2240

TTCCCCCAGGAGGGGGCTCAAACCCCCGCTCTGTGCCCTTTGAATACTACAGAATAAGAAAGGTTAAG

E2-P2A-Erns-P2A-Cap-P2A-Rep.seq(1>3645) → TTCCCCCAGGAGGGGGCTCAAACCCCCGCTCTGTGCCCTTTGAATACTACAGAATAAGAAAGGTTAAG

Y8847-3A(HZ275GN).1HZ275.7P_A08.scf(1>758) →

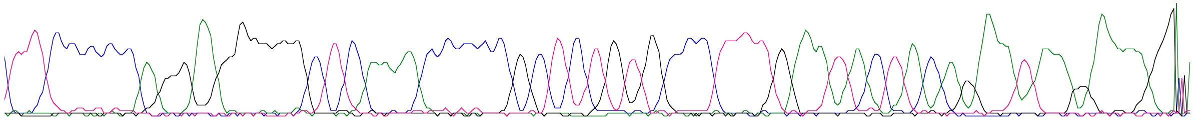

TTCCCCCAGGAGGGGGCTCAAACCCCCGCTCTGTGCCCTTTGAATACTACAGAATAAGAAAGG

Y8847-3A(HZ275GN).1HZ275.6P_H07.scf(1>760) ←

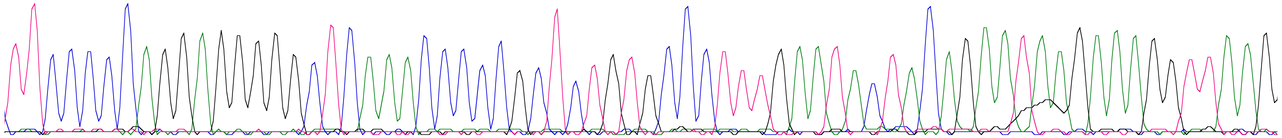

TTCCCCCAGGAGGGGGCTCAAACCCCCGCTCTGTGCCCTTTGAATACTACAGAATAAGAAAGGTTAAG

Y8847-3A(HZ275GN).1HZ275.9P_C08.scf(1>787) →

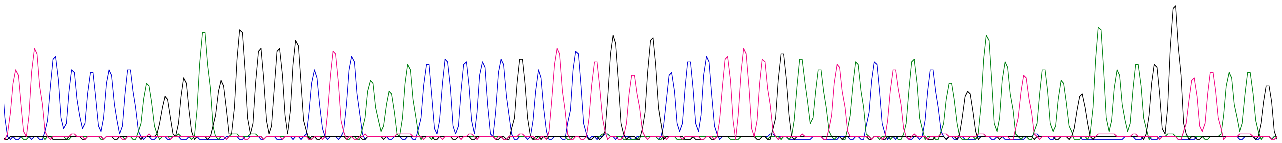

TTCCCCCAGGAGGGGGCTCAAACCCCCGCTCTGTGCCCTTTGAATACTACAGAATAAGAAAGGTTAAG

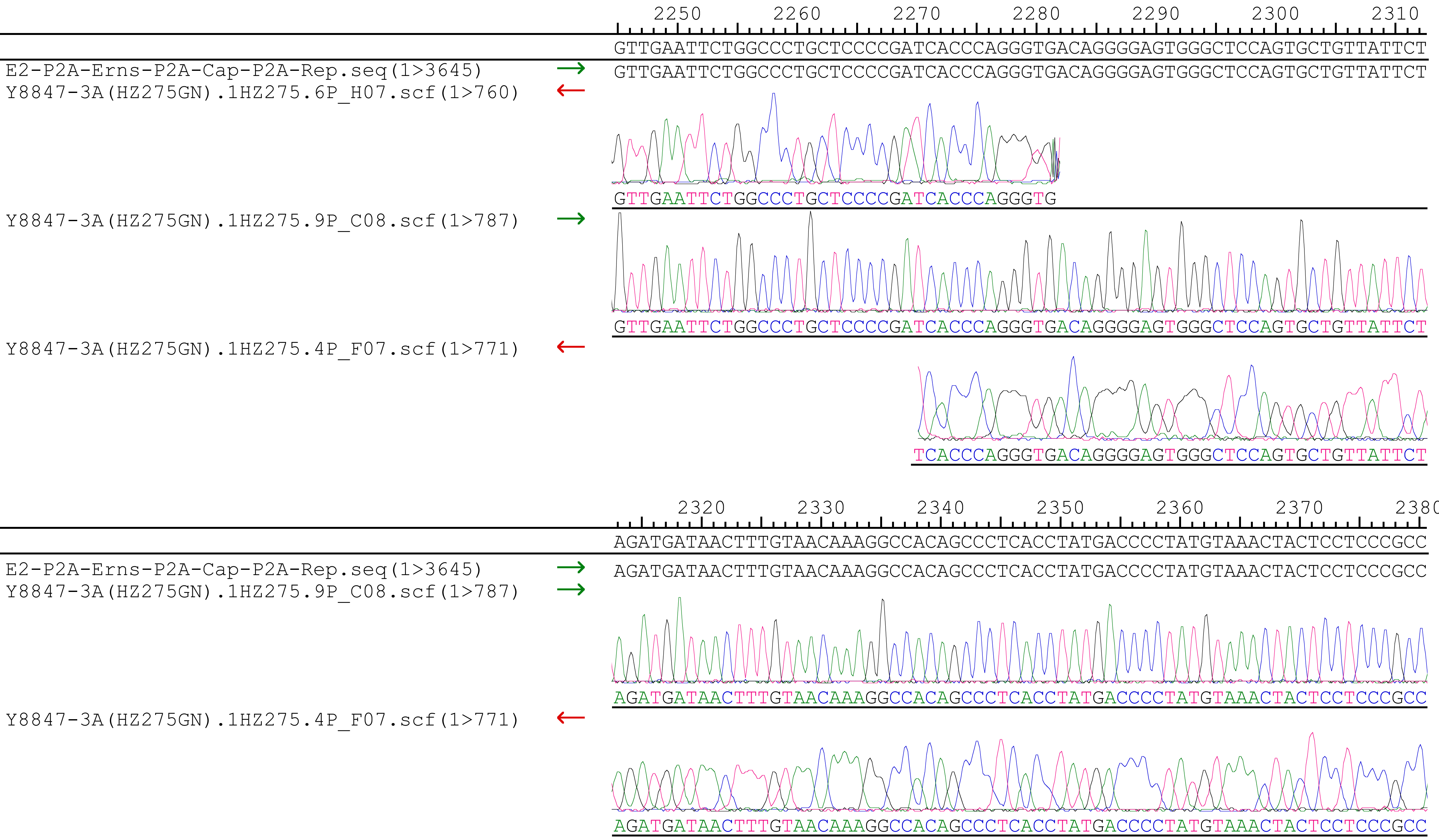
2250 2260 2270 2280 2290 2300 2310
GTTGAATTCTGGCCCTGCTCCCCGATCACCCAGGGTGACAGGGGAGTGGGCTCCAGTGCTGTTATTCT
E2-P2A-Erns-P2A-Cap-P2A-Rep.seq(1>3645)
GTTGAATTCTGGCCCTGCTCCCCGATCACCCAGGGTGACAGGGGAGTGGGCTCCAGTGCTGTTATTCT
Y8847-3A(HZ275GN).1HZ275.6P_H07.scf(1>760)
GTTGAATTCTGGCCCTGCTCCCCGATCACCCAGGGTG
Y8847-3A(HZ275GN).1HZ275.9P_C08.scf(1>787)
GTTGAATTCTGGCCCTGCTCCCCGATCACCCAGGGTGACAGGGGAGTGGGCTCCAGTGCTGTTATTCT
Y8847-3A(HZ275GN).1HZ275.4P_F07.scf(1>771)
TCACCCAGGGTGACAGGGGAGTGGGCTCCAGTGCTGTTATTCT
2320 2330 2340 2350 2360 2370 2380
AGATGATAACTTTGTAACAAAGGCCACAGCCCTCACCTATGACCCCTATGTAAACTACTCCTCCCGCC
E2-P2A-Erns-P2A-Cap-P2A-Rep.seq(1>3645)
AGATGATAACTTTGTAACAAAGGCCACAGCCCTCACCTATGACCCCTATGTAAACTACTCCTCCCGCC
Y8847-3A(HZ275GN).1HZ275.9P_C08.scf(1>787)
AGATGATAACTTTGTAACAAAGGCCACAGCCCTCACCTATGACCCCTATGTAAACTACTCCTCCCGCC
Y8847-3A(HZ275GN).1HZ275.4P_F07.scf(1>771)
AGATGATAACTTTGTAACAAAGGCCACAGCCCTCACCTATGACCCCTATGTAAACTACTCCTCCCGCC

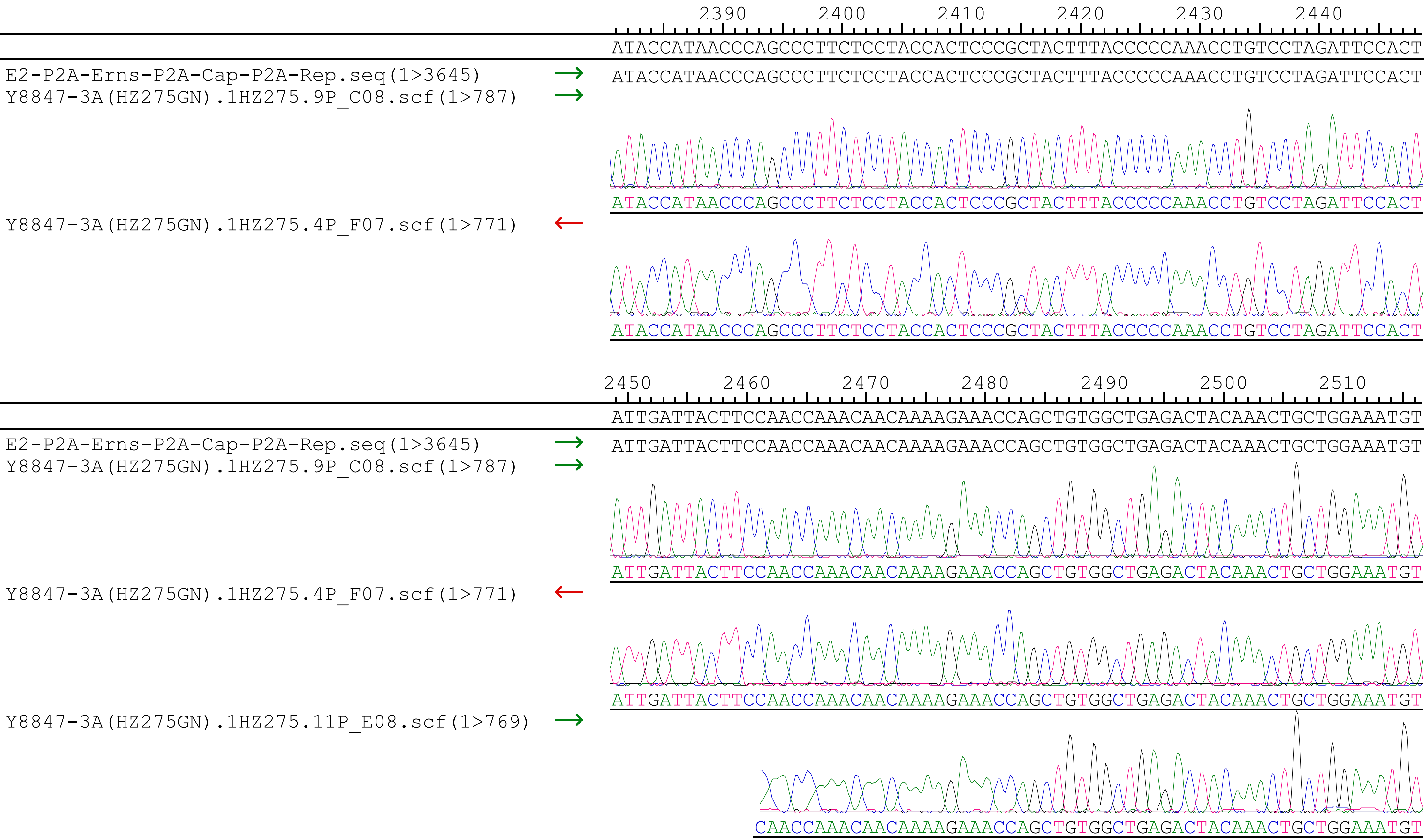
2390 2400 2410 2420 2430 2440
ATACCATAACCCAGCCCTTCTCCTACCACTCCCGCTACTTTACCCCCAAACCTGTCCTAGATTCCACT
E2-P2A-Erns-P2A-Cap-P2A-Rep.seq(1>3645)
ATACCATAACCCAGCCCTTCTCCTACCACTCCCGCTACTTTACCCCCAAACCTGTCCTAGATTCCACT
Y8847-3A(HZ275GN).1HZ275.9P_C08.scf(1>787)
ATACCATAACCCAGCCCTTCTCCTACCACTCCCGCTACTTTACCCCCAAACCTGTCCTAGATTCCACT
Y8847-3A(HZ275GN).1HZ275.4P_F07.scf(1>771)
ATACCATAACCCAGCCCTTCTCCTACCACTCCCGCTACTTTACCCCCAAACCTGTCCTAGATTCCACT
2450 2460 2470 2480 2490 2500 2510
ATTGATTACTTCCAACCAAACAACAAAAGAAACCAGCTGTGGCTGAGACTACAAACTGCTGGAAATGT
E2-P2A-Erns-P2A-Cap-P2A-Rep.seq(1>3645)
ATTGATTACTTCCAACCAAACAACAAAAGAAACCAGCTGTGGCTGAGACTACAAACTGCTGGAAATGT
Y8847-3A(HZ275GN).1HZ275.9P_C08.scf(1>787)
ATTGATTACTTCCAACCAAACAACAAAAGAAACCAGCTGTGGCTGAGACTACAAACTGCTGGAAATGT
Y8847-3A(HZ275GN).1HZ275.4P_F07.scf(1>771)
ATTGATTACTTCCAACCAAACAACAAAAGAAACCAGCTGTGGCTGAGACTACAAACTGCTGGAAATGT
Y8847-3A(HZ275GN).1HZ275.11P_E08.scf(1>769)
CAACCAAACAACAAAAGAAACCAGCTGTGGCTGAGACTACAAACTGCTGGAAATGT

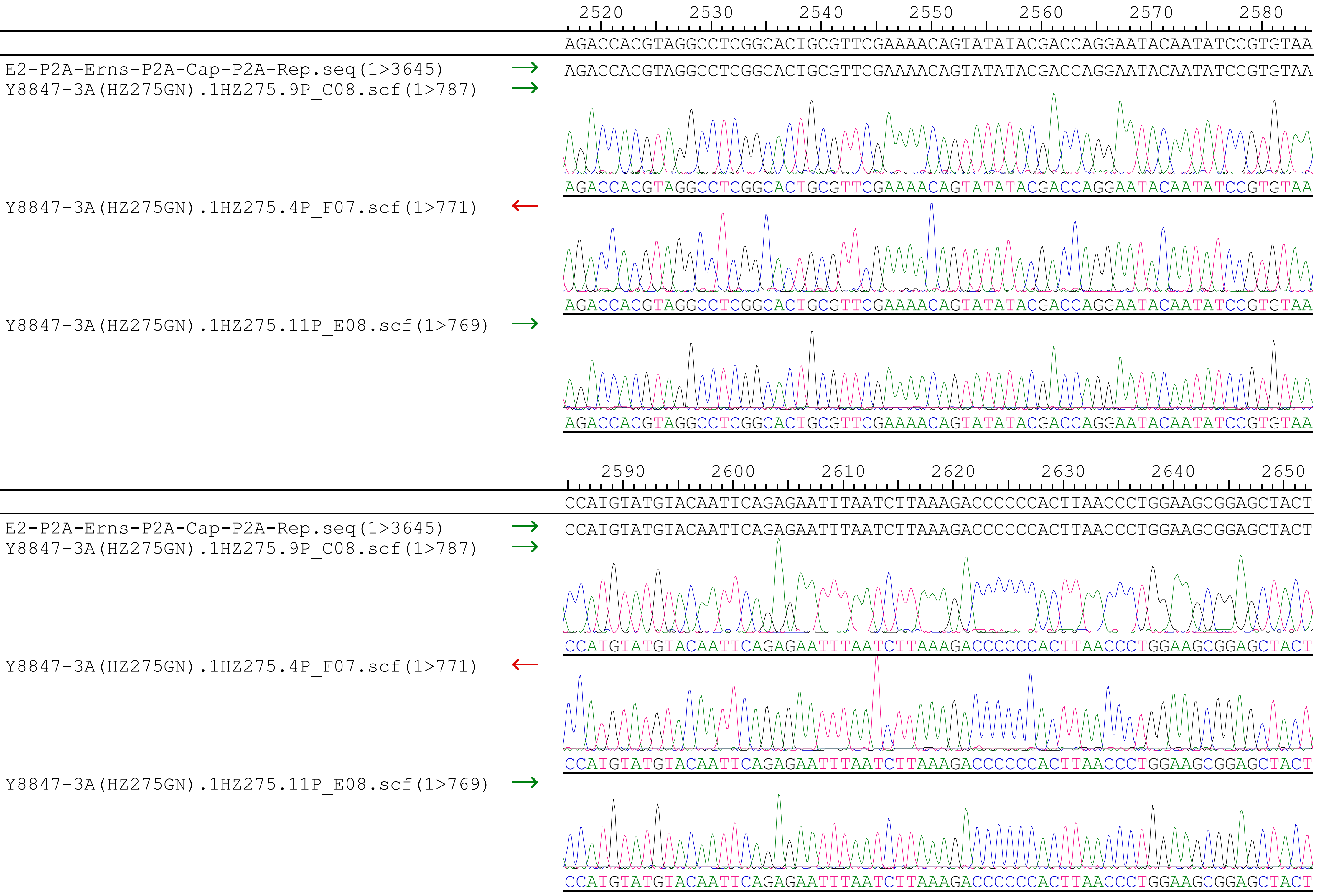
2520 2530 2540 2550 2560 2570 2580
AGACCACGTAGGCCTCGGCACTGCGTTCGAAAACAGTATATACGACCAGGAATACAATATCCGTGTAA
E2-P2A-Erns-P2A-Cap-P2A-Rep.seq(1>3645)
AGACCACGTAGGCCTCGGCACTGCGTTCGAAAACAGTATATACGACCAGGAATACAATATCCGTGTAA
Y8847-3A(HZ275GN).1HZ275.9P_C08.scf(1>787)
AGACCACGTAGGCCTCGGCACTGCGTTCGAAAACAGTATATACGACCAGGAATACAATATCCGTGTAA
Y8847-3A(HZ275GN).1HZ275.4P_F07.scf(1>771)
AGACCACGTAGGCCTCGGCACTGCGTTCGAAAACAGTATATACGACCAGGAATACAATATCCGTGTAA
Y8847-3A(HZ275GN).1HZ275.11P_E08.scf(1>769)
AGACCACGTAGGCCTCGGCACTGCGTTCGAAAACAGTATATACGACCAGGAATACAATATCCGTGTAA
2590 2600 2610 2620 2630 2640 2650
CCATGTATGTACAATTCAGAGAATTTAATCTTAAAGACCCCCCACTTAACCCTGGAAGCGGAGCTACT
E2-P2A-Erns-P2A-Cap-P2A-Rep.seq(1>3645)
CCATGTATGTACAATTCAGAGAATTTAATCTTAAAGACCCCCCACTTAACCCTGGAAGCGGAGCTACT
Y8847-3A(HZ275GN).1HZ275.9P_C08.scf(1>787)
CCATGTATGTACAATTCAGAGAATTTAATCTTAAAGACCCCCCACTTAACCCTGGAAGCGGAGCTACT
Y8847-3A(HZ275GN).1HZ275.4P_F07.scf(1>771)
CCATGTATGTACAATTCAGAGAATTTAATCTTAAAGACCCCCCACTTAACCCTGGAAGCGGAGCTACT
Y8847-3A(HZ275GN).1HZ275.11P_E08.scf(1>769)
CCATGTATGTACAATTCAGAGAATTTAATCTTAAAGACCCCCCACTTAACCCTGGAAGCGGAGCTACT

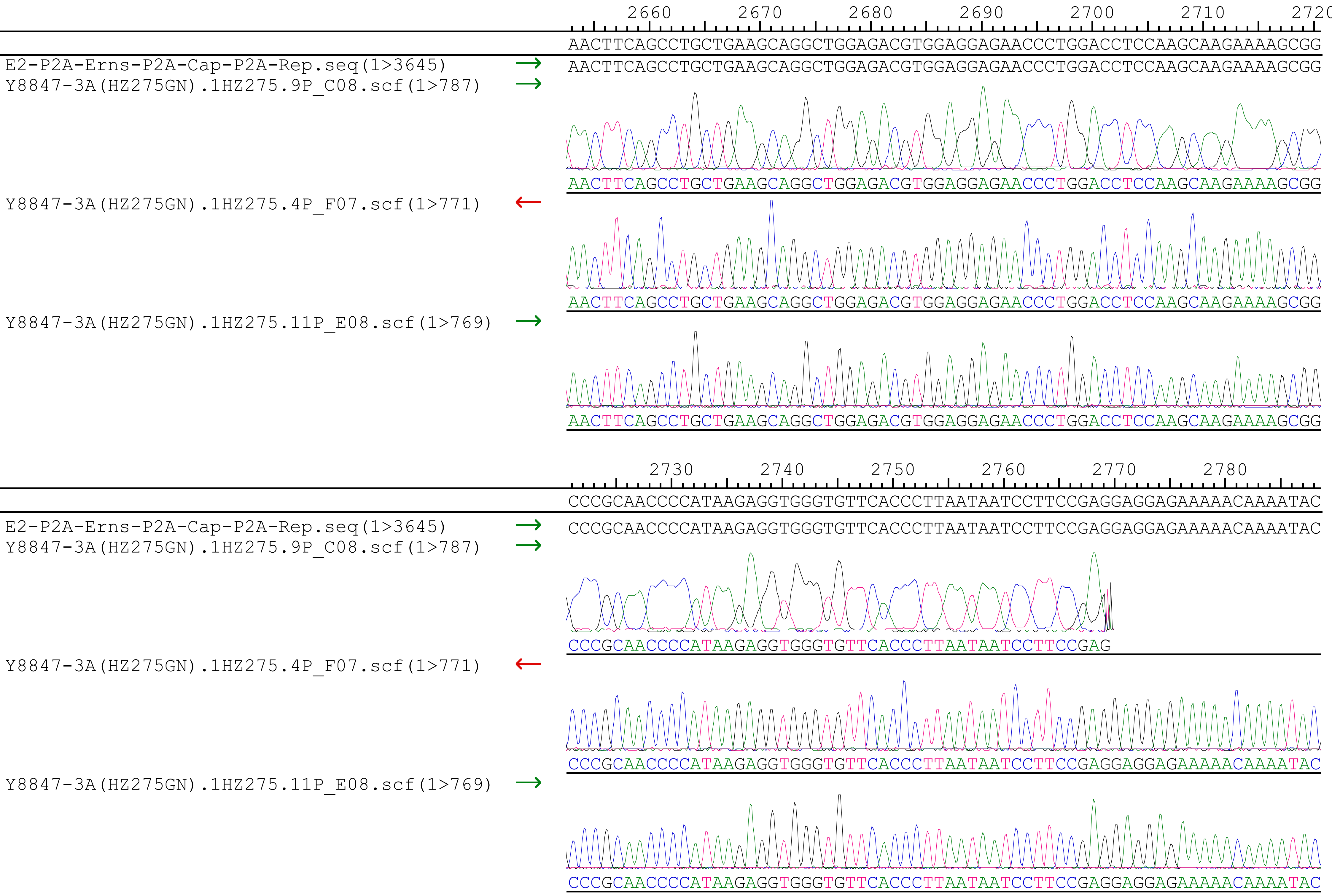
2660 2670 2680 2690 2700 2710 2720
AACTTCAGCCTGCTGAAGCAGGCTGGAGACGTGGAGGAGAACCCTGGACCTCCAAGCAAGAAAAGCGG
E2-P2A-Erns-P2A-Cap-P2A-Rep.seq(1>3645)
AACTTCAGCCTGCTGAAGCAGGCTGGAGACGTGGAGGAGAACCCTGGACCTCCAAGCAAGAAAAGCGG
Y8847-3A(HZ275GN).1HZ275.9P_C08.scf(1>787)
AACTTCAGCCTGCTGAAGCAGGCTGGAGACGTGGAGGAGAACCCTGGACCTCCAAGCAAGAAAAGCGG
Y8847-3A(HZ275GN).1HZ275.4P_F07.scf(1>771)
AACTTCAGCCTGCTGAAGCAGGCTGGAGACGTGGAGGAGAACCCTGGACCTCCAAGCAAGAAAAGCGG
Y8847-3A(HZ275GN).1HZ275.11P_E08.scf(1>769)
AACTTCAGCCTGCTGAAGCAGGCTGGAGACGTGGAGGAGAACCCTGGACCTCCAAGCAAGAAAAGCGG
2730 2740 2750 2760 2770 2780
CCCGCAACCCCATAAGAGGTGGGTGTTCACCCTTAATAATCCTTCCGAGGAGGAGAAAAACAAAATAC
E2-P2A-Erns-P2A-Cap-P2A-Rep.seq(1>3645)
CCCGCAACCCCATAAGAGGTGGGTGTTCACCCTTAATAATCCTTCCGAGGAGGAGAAAAACAAAATAC
Y8847-3A(HZ275GN).1HZ275.9P_C08.scf(1>787)
CCCGCAACCCCATAAGAGGTGGGTGTTCACCCTTAATAATCCTTCCGAG
Y8847-3A(HZ275GN).1HZ275.4P_F07.scf(1>771)
CCCGCAACCCCATAAGAGGTGGGTGTTCACCCTTAATAATCCTTCCGAGGAGGAGAAAAACAAAATAC
Y8847-3A(HZ275GN).1HZ275.11P_E08.scf(1>769)
CCCGCAACCCCATAAGAGGTGGGTGTTCACCCTTAATAATCCTTCCGAGGAGGAGAAAAACAAAATAC

2790 2800 2810 2820 2830 2840 2850

GGGAGCTTCCAATCTCCCTTTTTGATTATTTTGTTTGCGGAGAGGAAGGTTTGGAAGAGGGTAGAACT

E2-P2A-Erns-P2A-Cap-P2A-Rep.seq(1>3645) → GGGAGCTTCCAATCTCCCTTTTTGATTATTTTGTTTGCGGAGAGGAAGGTTTGGAAGAGGGTAGAACT

Y8847-3A(HZ275GN).1HZ275.4P_F07.scf(1>771) ←

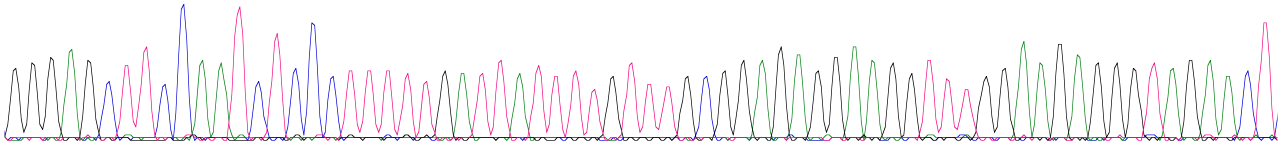

GGGAGCTTCCAATCTCCCTTTTTGATTATTTTGTTTGCGGAGAGGAAGGTTTGGAAGAGGGTAGAACT

Y8847-3A(HZ275GN).1HZ275.11P_E08.scf(1>769) →

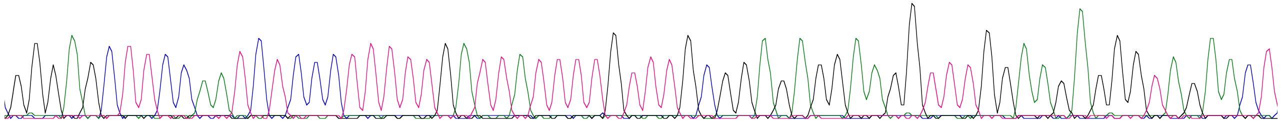

GGGAGCTTCCAATCTCCCTTTTTGATTATTTTGTTTGCGGAGAGGAAGGTTTGGAAGAGGGTAGAACT

2860 2870 2880 2890 2900 2910 2920

CCTCACCTCCAGGGGTTTGCGAATTTTGCTAAGAAGCAGACTTTTAACAAGGTGAAGTGGTATTTTGG

E2-P2A-Erns-P2A-Cap-P2A-Rep.seq(1>3645) → CCTCACCTCCAGGGGTTTGCGAATTTTGCTAAGAAGCAGACTTTTAACAAGGTGAAGTGGTATTTTGG

Y8847-3A(HZ275GN).1HZ275.4P_F07.scf(1>771) ←

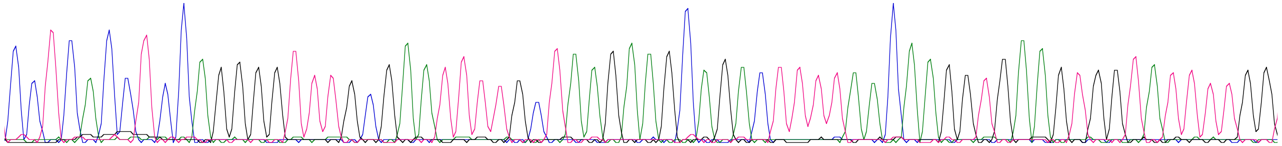

CCTCACCTCCAGGGGTTTGCGAATTTTGCTAAGAAGCAGACTTTTAACAAGGTGAAGTGGTATTTTGG

Y8847-3A(HZ275GN).1HZ275.11P_E08.scf(1>769) →

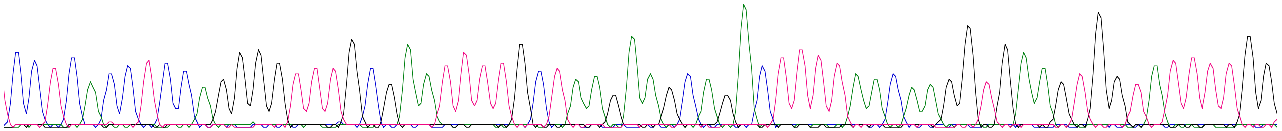

CCTCACCTCCAGGGGTTTGCGAATTTTGCTAAGAAGCAGACTTTTAACAAGGTGAAGTGGTATTTTGG

2930 2940 2950 2960 2970 2980 2990

TGCCCGCTGCCACATCGAGAAAGCGAAAGGAACCGACCAGCAGAATAAAGAATACTGCAGTAAAGAAG

E2-P2A-Erns-P2A-Cap-P2A-Rep.seq(1>3645) → TGCCCGCTGCCACATCGAGAAAGCGAAAGGAACCGACCAGCAGAATAAAGAATACTGCAGTAAAGAAG

Y8847-3A(HZ275GN).1HZ275.4P_F07.scf(1>771) ←

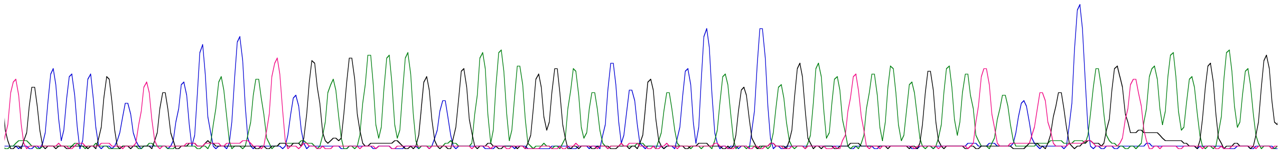

TGCCCGCTGCCACATCGAGAAAGCGAAAGGAACCGACCAGCAGAATAAAGAATACTGCAGTAAAGAAG

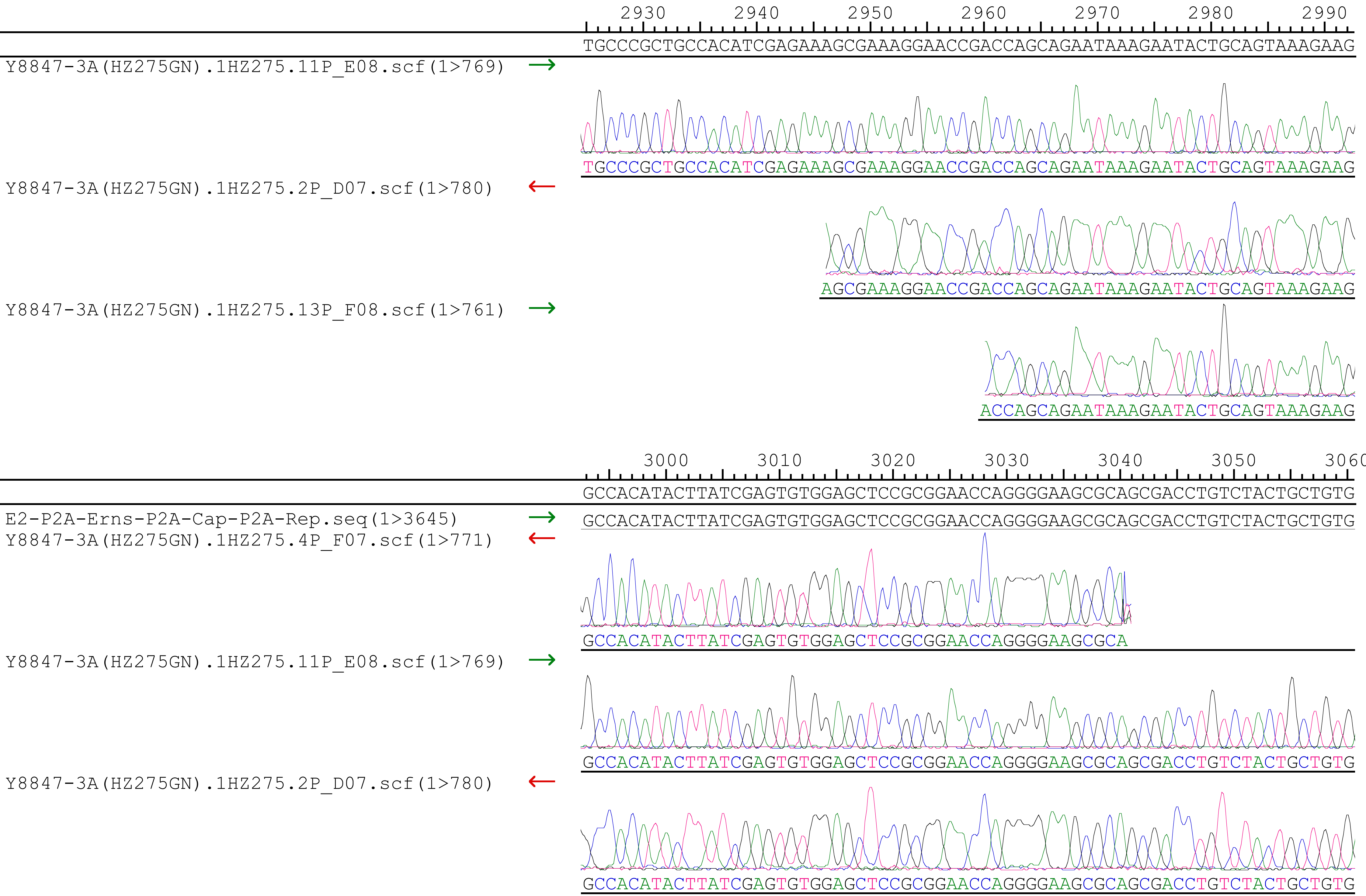

2930 2940 2950 2960 2970 2980 2990
TGCCCGCTGCCACATCGAGAAAGCGAAAGGAACCGACCAGCAGAATAAAGAATACTGCAGTAAAGAAG
Y8847-3A(HZ275GN).1HZ275.11P_E08.scf(1>769)
TGCCCGCTGCCACATCGAGAAAGCGAAAGGAACCGACCAGCAGAATAAAGAATACTGCAGTAAAGAAG
Y8847-3A(HZ275GN).1HZ275.2P_D07.scf(1>780)
AGCGAAAGGAACCGACCAGCAGAATAAAGAATACTGCAGTAAAGAAG
Y8847-3A(HZ275GN).1HZ275.13P_F08.scf(1>761)
ACCAGCAGAATAAAGAATACTGCAGTAAAGAAG
3000 3010 3020 3030 3040 3050 3060
GCCACATACTTATCGAGTGTGGAGCTCCGCGGAACCAGGGGAAGCGCAGCGACCTGTCTACTGCTGTG
E2-P2A-Erns-P2A-Cap-P2A-Rep.seq(1>3645)
GCCACATACTTATCGAGTGTGGAGCTCCGCGGAACCAGGGGAAGCGCAGCGACCTGTCTACTGCTGTG
Y8847-3A(HZ275GN).1HZ275.4P_F07.scf(1>771)
GCCACATACTTATCGAGTGTGGAGCTCCGCGGAACCAGGGGAAGCGCA
Y8847-3A(HZ275GN).1HZ275.11P_E08.scf(1>769)
GCCACATACTTATCGAGTGTGGAGCTCCGCGGAACCAGGGGAAGCGCAGCGACCTGTCTACTGCTGTG
Y8847-3A(HZ275GN).1HZ275.2P_D07.scf(1>780)
GCCACATACTTATCGAGTGTGGAGCTCCGCGGAACCAGGGGAAGCGCAGCGACCTGTCTACTGCTGTG

3000 3010 3020 3030 3040 3050 [illegible]

GCCACATACTTATCGAGTGTGGAGCTCCGCGGAACCAGGGGAAGCGCAGCGACCTGTCTACTGCTGTG

Y8847-3A(HZ275GN).1HZ275.13P_F08.scf(1>761) →

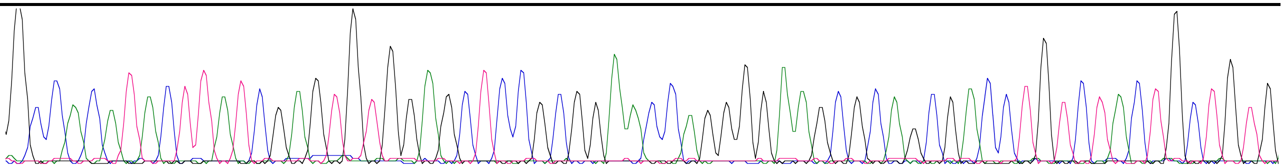

GCCACATACTTATCGAGTGTGGAGCTCCGCGGAACCAGGGGAAGCGCAGCGACCTGTCTACTGCTGTG

3070 3080 3090 3100 3110 3120

AGTACCCTTTTGGAGACGGGGTCTTTGGTGACTGTAGCCGAGCAGTTCCCTGTAACGTATGTGAGAAA

E2-P2A-Erns-P2A-Cap-P2A-Rep.seq(1>3645) → AGTACCCTTTTGGAGACGGGGTCTTTGGTGACTGTAGCCGAGCAGTTCCCTGTAACGTATGTGAGAAA

Y8847-3A(HZ275GN).1HZ275.11P_E08.scf(1>769) →

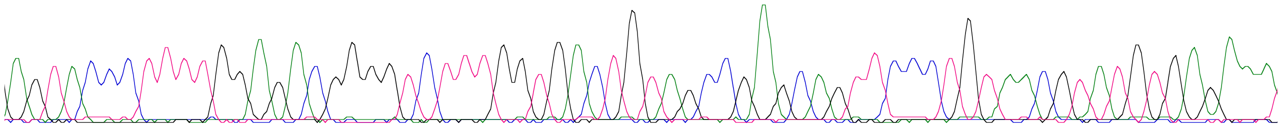

AGTACCCTTTTGGAGACGGGGTCTTTGGTGACTGTAGCCGAGCAGTTCCCTGTAACGTATGTGAGAAA

Y8847-3A(HZ275GN).1HZ275.2P_D07.scf(1>780) ←

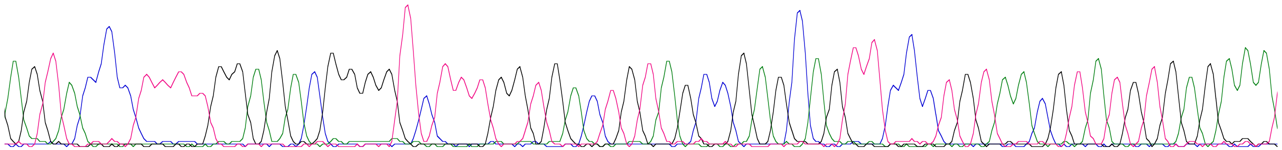

AGTACCCTTTTGGAGACGGGGTCTTTGGTGACTGTAGCCGAGCAGTTCCCTGTAACGTATGTGAGAAA

Y8847-3A(HZ275GN).1HZ275.13P_F08.scf(1>761) →

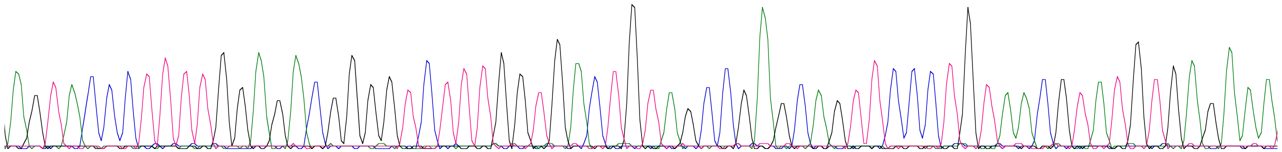

AGTACCCTTTTGGAGACGGGGTCTTTGGTGACTGTAGCCGAGCAGTTCCCTGTAACGTATGTGAGAAA

3130 3140 3150 3160 3170 3180 3190

TTTCCGCGGGCTGGCTGAACTTTTGAAAGTGAGCGGGAAGATGCAGCAGCGTGATTGGAAGACAGCTG

E2-P2A-Erns-P2A-Cap-P2A-Rep.seq(1>3645) → TTTCCGCGGGCTGGCTGAACTTTTGAAAGTGAGCGGGAAGATGCAGCAGCGTGATTGGAAGACAGCTG

Y8847-3A(HZ275GN).1HZ275.11P_E08.scf(1>769) →

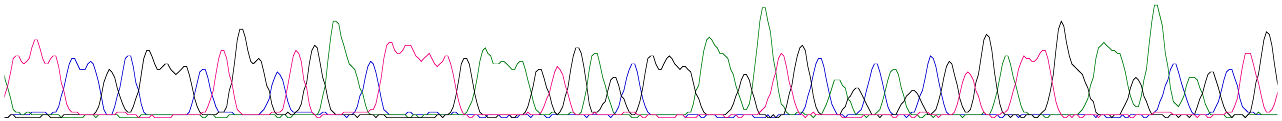

TTTCCGCGGGCTGGCTGAACTTTTGAAAGTGAGCGGGAAGATGCAGCAGCGTGATTGGAAGACAGCTG

3130 3140 3150 3160 3170 3180 3190

TTTCCGCGGGCTGGCTGAACTTTTGAAAGTGAGCGGGAAGATGCAGCAGCGTGATTGGAAGACAGCTG

Y8847-3A(HZ275GN).1HZ275.2P_D07.scf(1>780) ←

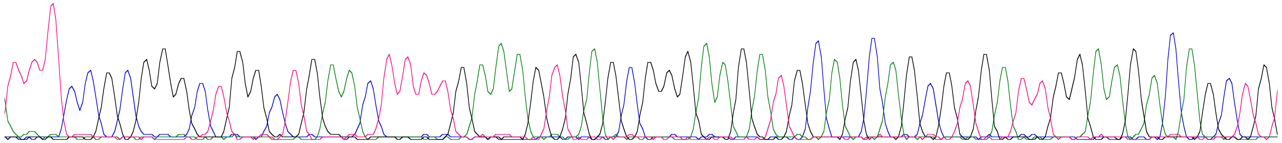

TTTCCGCGGGCTGGCTGAACTTTTGAAAGTGAGCGGGAAGATGCAGCAGCGTGATTGGAAGACAGCTG

Y8847-3A(HZ275GN).1HZ275.13P_F08.scf(1>761) →

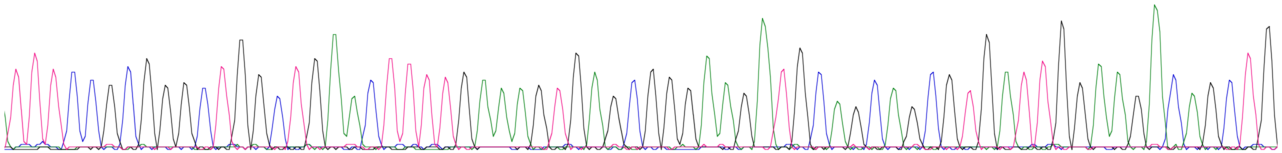

TTTCCGCGGGCTGGCTGAACTTTTGAAAGTGAGCGGGAAGATGCAGCAGCGTGATTGGAAGACAGCTG

3200 3210 3220 3230 3240 3250 3260

TACACGTCATAGTGGGCCCGCCCGGTTGTGGGAAGAGCCAGTGGGCCCGTAATTTTGCTGAGCCTAGC

E2-P2A-Erns-P2A-Cap-P2A-Rep.seq(1>3645) → TACACGTCATAGTGGGCCCGCCCGGTTGTGGGAAGAGCCAGTGGGCCCGTAATTTTGCTGAGCCTAGC

Y8847-3A(HZ275GN).1HZ275.11P_E08.scf(1>769) →

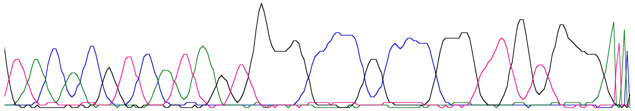

TACACGTCATAGTGGGCCCGCCCGGTTGTGGGA

Y8847-3A(HZ275GN).1HZ275.2P_D07.scf(1>780) ←

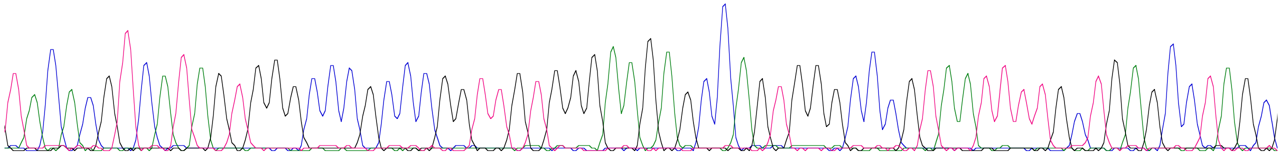

TACACGTCATAGTGGGCCCGCCCGGTTGTGGGAAGAGCCAGTGGGCCCGTAATTTTGCTGAGCCTAGC

Y8847-3A(HZ275GN).1HZ275.13P_F08.scf(1>761) →

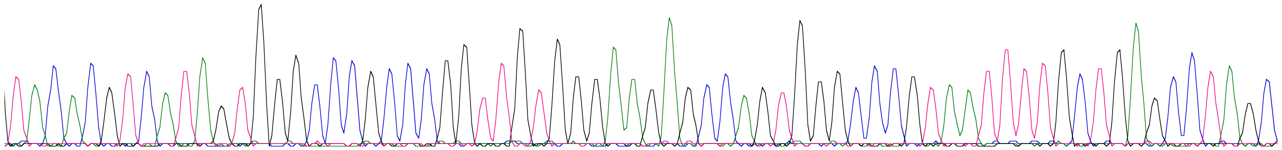

TACACGTCATAGTGGGCCCGCCCGGTTGTGGGAAGAGCCAGTGGGCCCGTAATTTTGCTGAGCCTAGC

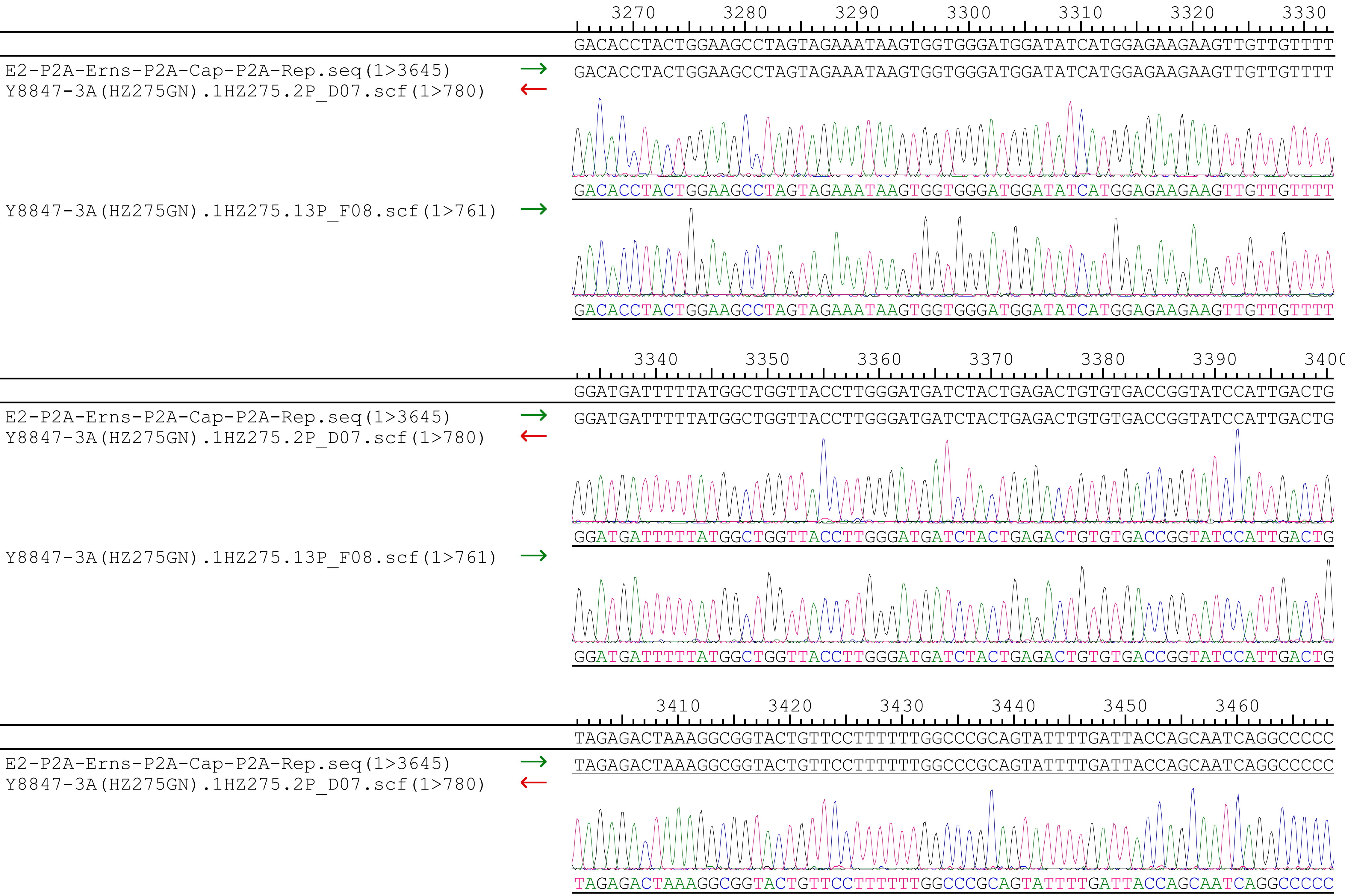
3270 3280 3290 3300 3310 3320 3330
GACACCTACTGGAAGCCTAGTAGAAATAAGTGGTGGGATGGATATCATGGAGAAGAAGTTGTTGTTTT
E2-P2A-Erns-P2A-Cap-P2A-Rep.seq(1>3645)
Y8847-3A(HZ275GN).1HZ275.2P_D07.scf(1>780)
Y8847-3A(HZ275GN).1HZ275.13P_F08.scf(1>761)
3340 3350 3360 3370 3380 3390 3400
GGATGATTTTTATGGCTGGTTACCTTGGGATGATCTACTGAGACTGTGTGACCGGTATCCATTGACTG
3410 3420 3430 3440 3450 3460
TAGAGACTAAAGGCGGTACTGTTCCTTTTTTGGCCCGCAGTATTTTGATTACCAGCAATCAGGCCCCC

3410 3420 3430 3440 3450 3460

TAGAGACTAAAGGCGGTACTGTTCCTTTTTTGGCCCGCAGTATTTTGATTACCAGCAATCAGGCCCCC

Y8847-3A(HZ275GN).1HZ275.13P_F08.scf(1>761) →

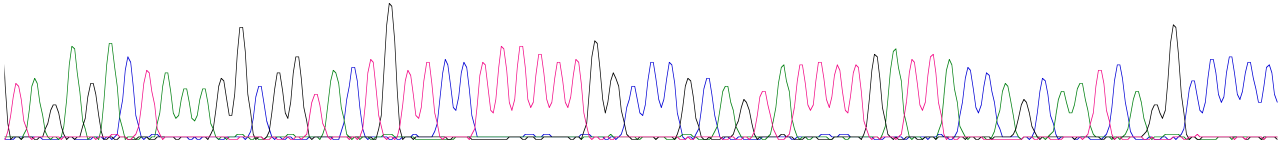

TAGAGACTAAAGGCGGTACTGTTCCTTTTTTGGCCCGCAGTATTTTGATTACCAGCAATCAGGCCCCC

3470 3480 3490 3500 3510 3520 3530

CAGGAATGGTACTCCTCAACTGCTGTCCCAGCTGTAGAAGCTCTCTATCGGAGGATTACTACTTTGCA

E2-P2A-Erns-P2A-Cap-P2A-Rep.seq(1>3645) → CAGGAATGGTACTCCTCAACTGCTGTCCCAGCTGTAGAAGCTCTCTATCGGAGGATTACTACTTTGCA

Y8847-3A(HZ275GN).1HZ275.2P_D07.scf(1>780) ←

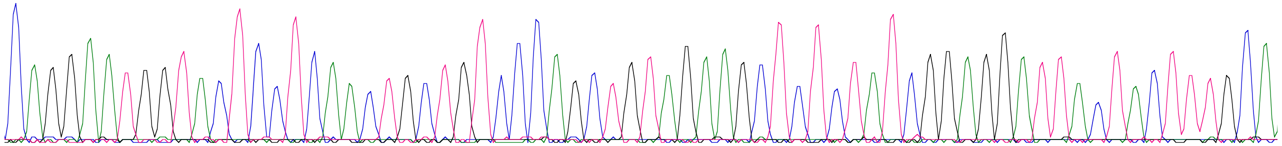

CAGGAATGGTACTCCTCAACTGCTGTCCCAGCTGTAGAAGCTCTCTATCGGAGGATTACTACTTTGCA

Y8847-3A(HZ275GN).1HZ275.13P_F08.scf(1>761) →

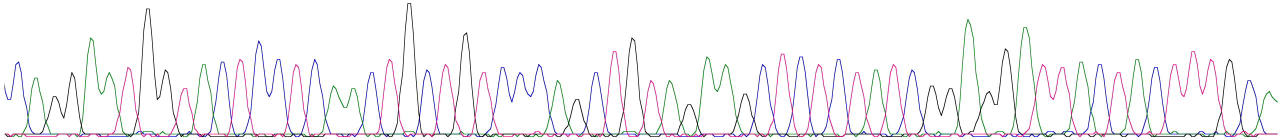

CAGGAATGGTACTCCTCAACTGCTGTCCCAGCTGTAGAAGCTCTCTATCGGAGGATTACTACTTTGCA

3540 3550 3560 3570 3580 3590 3600

ATTTTGGAAGACTGCTGGAGAACAATCCACGGAGGTACCCGAAGGCCGATTTGAAGCAGTGGACCCAC

E2-P2A-Erns-P2A-Cap-P2A-Rep.seq(1>3645) → ATTTTGGAAGACTGCTGGAGAACAATCCACGGAGGTACCCGAAGGCCGATTTGAAGCAGTGGACCCAC

Y8847-3A(HZ275GN).1HZ275.2P_D07.scf(1>780) ←

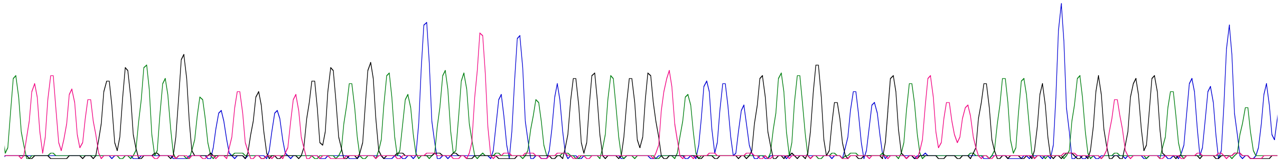

ATTTTGGAAGACTGCTGGAGAACAATCCACGGAGGTACCCGAAGGCCGATTTGAAGCAGTGGACCCAC

Y8847-3A(HZ275GN).1HZ275.13P_F08.scf(1>761) →

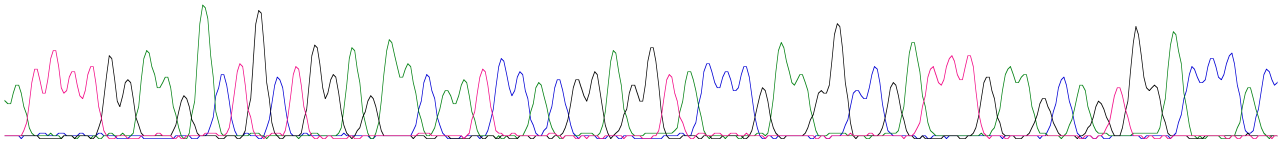

ATTTTGGAAGACTGCTGGAGAACAATCCACGGAGGTACCCGAAGGCCGATTTGAAGCAGTGGACCCAC

3610 3620 3630 3640 3650 3660 3670

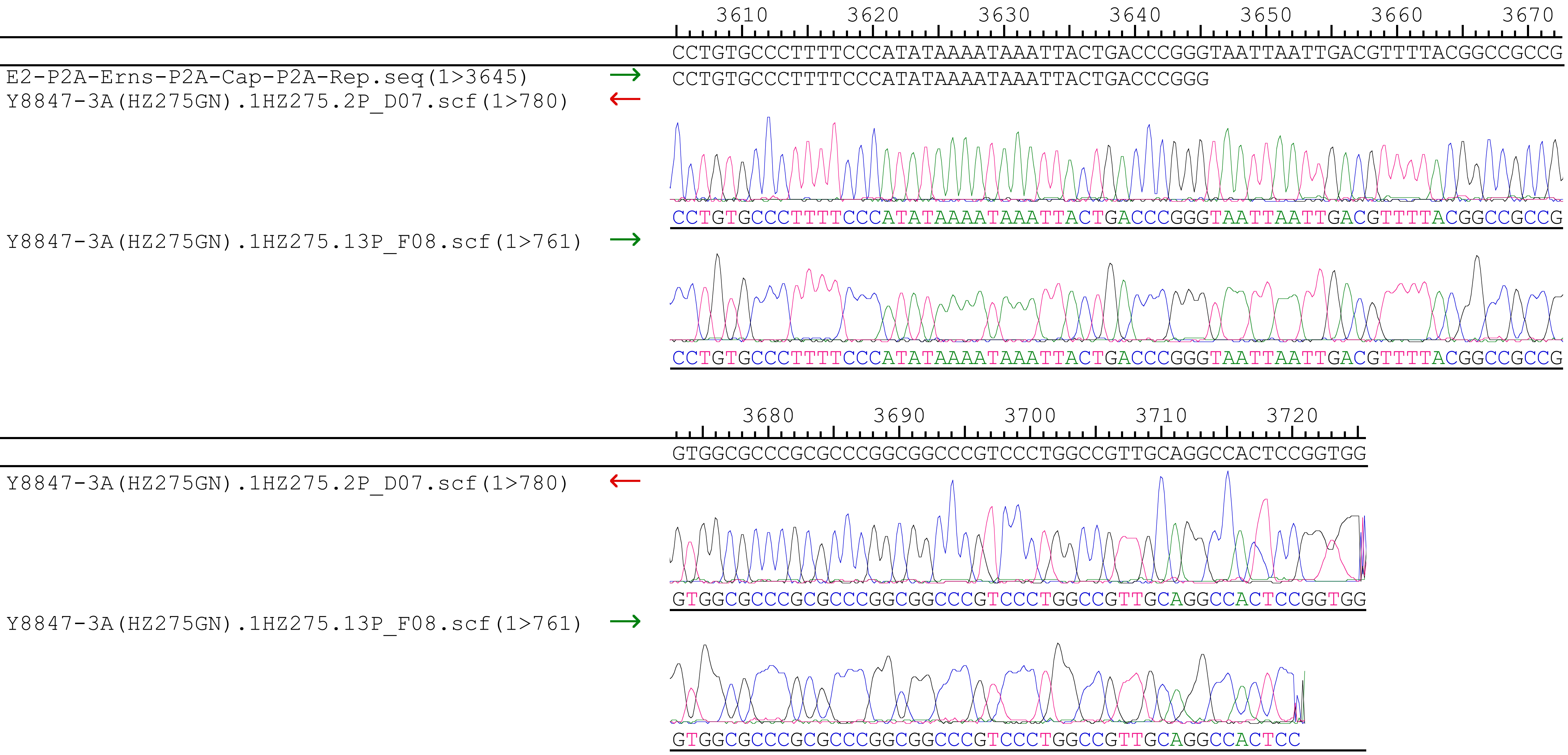
3610 3620 3630 3640 3650 3660 3670
CCTGTGCCCTTTTCCCATATAAAATAAATTACTGACCCGGGTAATTAATTGACGTTTTACGGCCGCCG
E2-P2A-Erns-P2A-Cap-P2A-Rep.seq(1>3645)
CCTGTGCCCTTTTCCCATATAAAATAAATTACTGACCCGGG
Y8847-3A(HZ275GN).1HZ275.2P_D07.scf(1>780)
CCTGTGCCCTTTTCCCATATAAAATAAATTACTGACCCGGGTAATTAATTGACGTTTTACGGCCGCCG
Y8847-3A(HZ275GN).1HZ275.13P_F08.scf(1>761)
CCTGTGCCCTTTTCCCATATAAAATAAATTACTGACCCGGGTAATTAATTGACGTTTTACGGCCGCCG
3680 3690 3700 3710 3720
GTGGCGCCCGCGCCCGGCGGCCCGTCCCTGGCCGTTGCAGGCCACTCCGGTGG
Y8847-3A(HZ275GN).1HZ275.2P_D07.scf(1>780)
GTGGCGCCCGCGCCCGGCGGCCCGTCCCTGGCCGTTGCAGGCCACTCCGGTGG
Y8847-3A(HZ275GN).1HZ275.13P_F08.scf(1>761)
GTGGCGCCCGCGCCCGGCGGCCCGTCCCTGGCCGTTGCAGGCCACTCC
